# Linking neurological status to functional outcomes in spinal cord injury: a multi-class, task-specific approach

**DOI:** 10.1186/s42490-026-00110-1

**Published:** 2026-04-15

**Authors:** Miklovana Tuci, Lena Steck, Louis P. Lukas, Olga Taran, Ruediger Rupp, Norbert Weidner, Martin Schubert, Frank Röhrich, Josina Waldmann, Yorck B. Kalke, Rainer Abel, Doris Maier, Thomas Liebscher, Björn Zörner, Armin Curt, Marc Bolliger, Sarah C. Brüningk, Catherine R. Jutzeler

**Affiliations:** 1 Department of Health Sciences and Technology (D-HEST), ETH Zurich, Zürich Switzerland; 2 SIB Swiss Institute of Bioinformatics, Lausanne, Switzerland; 3 Spinal Cord Injury Center, University Hospital Balgrist, University of Zurich, Zürich, Switzerland; 4 Spinal Cord Injury Center, Heidelberg University Hospital, Heidelberg, Germany; 5 Spinal Cord Injury Center, BG Klinikum Bergmannstrost Halle, Halle, Germany; 6 Orthopädische Klinik, Hessisch Lichtenau, Germany; 7 Spinal Cord Injury Center Orthopaedic Department, Ulm University, Ulm, Germany; 8 Spinal Cord Injury Center, Bayreuth, Germany; 9 Spinal Cord Injury Center, Trauma Center Murnau, Murnau, Germany; 10 Treatment Centre for Spinal Cord Injuries, Trauma Hospital Berlin, Berlin, Germany; 11 Swiss Paraplegic Centre, Nottwil, Switzerland; 12 Faculty of Health Sciences and Medicine Health and Rehabilitation Sciences, University of Lucerne, Lucerne, Switzerland; 13 Department of Radiation Oncology, Inselspital, Bern University Hospital and University of Bern, Bern, Switzerland; 14 Medical Faculty Heidelberg, Heidelberg University, Heidelberg, Germany

**Keywords:** Spinal cord injury, Functional outcomes, Neurological status, Multi-class analysis, Task-specific, Machine learning

## Abstract

**Background:**

Spinal cord injury (SCI) causes long-term neurological deficits resulting in functional disabilities. While longitudinal recovery patterns of sensorimotor outcomes after SCI have been studied, few analyses have applied machine learning to systematically model the relationship between neurological impairments and functional independence at different post-injury phases.

**Methods:**

This study compared ordinal and nominal classification models predicting functional independence from sensorimotor status cross-sectionally. Inputs included motor and sensory scores from the International Standards for Neurological Classification of SCI, age, sex, and time since injury collected in the European Multicenter Study about SCI. Models were evaluated on a task from each domain of the Spinal Cord Independence Measure, namely grooming (self-care), bladder management (respiration and sphincter management), and indoor mobility (mobility). Analyses were stratified into early (≤ 40 days), intermediate (70–100 days), and late (> 182 days) post-injury phases. Models were ranked based on five evaluation metrics, and interpretability explored using Shapley Additive Explanations (SHAP).

**Results:**

Model accuracy improved over time (early phase: 46–71%, late phase: 50–85%), indicating that functional independence is more reliably determined from sensorimotor scores in later post-injury phases. Across all scenarios, random forest achieved the best overall performance (0.93 ± 0.03, averaged across different metrics). Ordinal models yielded fewer severe misclassifications compared to nominal models. Motor scores were stronger predictors than sensory scores, with lower limb function (L2–L4) strongly associated with mobility, voluntary anal contraction with bladder control, and upper limb function (C6, C8) with grooming ability, highlighting that models utilise known relationships.

**Conclusion:**

We show that both multiclass and ordinal models can accurately classify SCIM-based functional independence outcomes after SCI from neurological assessments at different time points post-injury. Ordinal approaches provide particular clinical value by minimizing severe misclassifications, a crucial advantage when distinguishing between functional independence classes that require fundamentally different care approaches. Interpretability analysis showed that the predictions are grounded in clinical knowledge. The developed models provide the basis for a modular prognostic framework, in which predicted ISNCSCI scores can be used to derive the most likely functional independence class, enabling a modular, computationally efficient and scalable approach to prediction in SCI care across a range of neurological and functional outcomes.

**Supplementary Information:**

The online version contains supplementary material available at 10.1186/s42490-026-00110-1.

## Background

Spinal cord injury (SCI) often results in permanent neurological impairment, significantly impacting daily functioning and quality of life [1]. The International Standards for Neurological Classification of Spinal Cord Injury (ISNCSCI) [2] offers a standardized classification of lesion level and severity, by assessing motor and sensory function, while independence in activities of daily living such as grooming or walking indoors are measured using the Spinal Cord Independence Measure (SCIM) [3]. Both assessments use ordinal scales to score individual motor and sensory function or task performance but commonly total sum scores of individual items are considered to evaluate rehabilitation outcomes.

A recent review showed that several studies addressed the prediction of long-term functional outcomes using statistical and machine learning models based on neurological assessments obtained early after injury [4], identifying specific myotomes as significant predictors of walking ability and SCIM self-care scores [5–7]. Correlation analyses have shown a strong association between total motor scores (MS) and total SCIM scores in individuals with cervical injuries (e.g., *r* = 0.63, *p* < 0.001, in tetraplegia) [8], though this relationship weakens in thoracic lesions [8–10]. 

However, most existing studies utilised regression models which predict sum scores [5, 6, 11–17], or dichotomized results for specific functional outcomes [18–33]. Both approaches compress complex functional information, either by aggregating scores across tasks involving different body regions (e.g., by predicting a total SCIM score) or abstracting detailed descriptions of function (e.g., walking function categorized as present or absent while corresponding SCIM items contain up to nine distinct levels), thereby potentially impairing interpretability of results and reducing sensitivity to clinically meaningful variation [34, 35]. Furthermore, the use of regression models treats the outcome to be predicted as continuous, which does not reflect the ordinal nature of many assessment scales like SCIM.

In addition to these limitations, the cross-sectional relationship between neurological impairments and functional independence remains insufficiently characterized [36]. Importantly, neurological recovery does not always translate into functional improvement [37], and functional gains may result from compensatory strategies which are only partly captured by standard neurological assessments, increasing the complexity of longitudinal predictions [38, 39]. These observations underscore the importance of comprehensively investigating how neurological and functional measures associated over the course of recovery after SCI. Importantly, identifying which key neurological assessments are most predictive of specific aspects of functional independence, and how these change over time, can reveal important targets for rehabilitation strategies. In this study, we systematically investigate the cross-sectional relationship between neurological status and functional independence at multiple time points following SCI. Using one of the largest datasets available for outcomes after SCI, the European Multicenter Study about SCI (EMSCI), we compare multi-class and ordinal classification models of varying complexity for associations with specific functional independence tasks assessed through SCIM. We hypothesize that distinct sets of neurological impairment patterns are associated with different functional tasks and that these key contributors vary across phases of recovery.

## Methods

### Study design and patient population selection

This study represents a secondary analysis of ISNCSCI and SCIM datasets from the EMSCI, as of 2023, an ongoing longitudinal observational study initiated in 2001 (ClinicalTrials.gov Identifier: NCT01571531, https://www.emsci.org, registered 02 April 2012). This dataset comprises patients with traumatic or ischemic spinal cord injuries. The study population spans a range of injury severities, including both complete and incomplete injuries as defined by the American Spinal Injury Association (ASIA) Impairment Scale (AIS; grades A–D), and includes cervical, thoracic, and lumbar injury levels. Demographic variables available for this analysis include age and sex. Detailed inclusion and exclusion criteria are defined in the EMSCI study protocol and are publicly available on the study website. The design and reporting of this study adhere to the STrengthening the Reporting of OBservational studies in Epidemiology (STROBE) guidelines for observational studies [40]. Three subsets of participants were defined according to the timing of their assessment: early (≤ 40 days after injury (DAI)), intermediate (70–100 DAI), and late (> 182 DAI). To reduce variability because of differences in the assessment time point, for each patient, we excluded observations in which the time between their ISNCSCI and SCIM assessments exceeded 7 days in the early and intermediate subsets, and 30 days in the late subset. Observations with inconsistencies between SCIM item 12 (mobility indoors) and walking test results were also excluded (Fig. S1). This study was conducted as a complete case analysis, excluding all records with missing data in any input or output features (including features marked as “not testable”). Additionally, cases with no defined AIS grades were not included.

### Input (independent) features

**Fig. 1:**
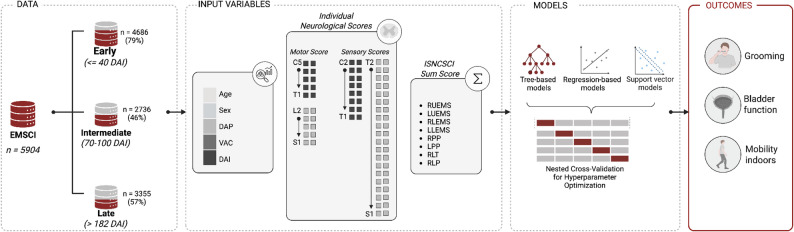
Analysis pipeline used for model training. The independence in three functional tasks were analysed across three data subsets, each representing a different injury stage, using two distinct feature sets describing the neurological status. Abbreviations: DAI, Days after injury; DAP, deep anal pressure; VAC, voluntary anal contractions; ISNCSCI, International Standards for the Neurological Classification of Spinal Cord Injury; RUEMS, right upper extremity motor score; LUEMS, left upper extremity motor score; RLEMS, right lower extremity motor score, LLEMS, left lower extremity motor score; RPP, right pinprick; LPP, left pin prick; RLT, right light touch; LLT, left light touch; SCIM, Spinal Cord Independence Measure

Figure 1 outlines the included input features: segmental motor and sensory scores (MS, SS) from the ISNCSCI assessment [2], including deep anal pressure (DAP) and voluntary anal contraction (VAC); timing of these assessments as days after injury (DAI); demographic features (age, sex). MS were evaluated in 10 key muscle groups of the upper and lower extremities, with each muscle group graded on a scale from 0 to 5, where 0 indicates total paralysis and 5 represents normal muscle function. The sensory scores were assessed for light touch and pinprick sensation across 28 dermatomes on each side of the body. Sensory testing was performed according to the ISNCSCI protocol, with light touch assessed using a cotton wisp and pinprick assessed using a disposable safety pin to evaluate sharp-dull discrimination. Each sensory point was rated on a scale from 0 to 2, with 0 indicating absent, 1 indicating altered sensation, and 2 representing normal sensory perception. In addition to segmental data, summary scores were also used as alternative input features in separate analyses. These included: right and left upper extremity motor scores (UEMS), right and left lower extremity motor scores (LEMS), and total light touch and pinprick scores for the right and left sides of the body. All input features were normalized to a (0, 1) range prior to model training.

### Outcome features

In this study, SCIM item scores were used as outcome variables in the classification models. The SCIM evaluates functional independence across three domains: self-care, respiration and sphincter management, and mobility [3]. While existing prediction models mostly use the total SCIM score to represent functional recovery after SCI [17, 41]. In line with existing work, we focused on three domains previously studied [21, 28, 30], represented by SCIM Item 4: Grooming, Item 6: Sphincter Management: Bladder, and Item 12: Mobility Indoors respectively. EMSCI includes SCIM II [42] and SCIM III [3], which differ slightly in the scoring of grooming and bladder management. To ensure compatibility across versions, these items were harmonized into corresponding classes (see Table S1). Furthermore, for the bladder management and mobility items classes with very few instances were merged into a single class to increase subset size and improve model stability (see Table S2). All selected SCIM items were ordinally encoded.

### Classification models

We compared standard and specialized machine learning models designed to analyse cross-sectional relationships between neurological assessments and functional independence measured within the same time window. Three widely used classification models were implemented using Python’s Scikit-Learn [43] library: logistic regression [44], random forest [45], and support vector machine (SVM) [46]. Each model was adjusted to account for imbalances in the dataset by giving more weight to less common outcome categories. As functional outcome scores in this study follow a natural order, we also used ordinal classification models designed for ranked outcomes. These included a cumulative link model (CLM) [47], and an ordinal random forest, both implemented in R. We further applied an ordered partitions (OP) method [48, 49], which decomposes *k* ordinal classes into *k-1* binary prediction tasks where each classifier determines whether the outcome exceeds a given threshold (e.g. is the outcome class ≤ 2 vs. > 2), with the final prediction corresponding to the highest threshold satisfied. This method was tested with both random forest and SVM. Training was conducted independently across all combinations of dataset subset (early, intermediate, late), input type (segmental vs. summary scores), and outcome feature (grooming, bladder management, mobility). This resulted in nine distinct classification tasks, referred to throughout as *scenarios*.

### Evaluation metrics and model selection

All models were assessed using five-fold cross-validation stratified by outcome and grouped by patient ID, so that all data from each patient remained within a single fold. Model performance was scored by accuracy, balanced accuracy, calculated as the average of the true positive rate (proportion of actual positive cases correctly identified) and true negative rate (proportion of actual negative cases correctly identified) to account for class imbalance, mean absolute error (MAE), macro-averaged MAE (averaging errors equally across classes), and quadratic weighted kappa (QWK) [50], which evaluates the agreement between predicted and true ordinal scores with greater penalty for larger disagreements. In this way we evaluate not only classification accuracy, but also the severity of prediction errors. These metrics were combined into a composite score using a simple additive weighting (SAW) [51], with equal weights. For each scenario, the performance of every model was calculated relative to the best-performing model, with the best performance for the respective scenario corresponding to 100%. These relative scores were averaged across scenarios for an overall performance ranking. Models were also ranked within each scenario. This approach was used to integrate multiple performance metrics to enable consistent comparison of models across multiple scenarios. Hyperparameter tuning was conducted using nested five-fold cross-validation, also stratified by class and grouped by patient ID. The hyperparameters tested are listed in Table S3. For nominal models, hyperparameters were optimized based on balanced accuracy. For ordinal models, the optimal optimization criterion was determined by systematically comparing the outer fold performance of three criteria, balanced accuracy, macro-averaged MAE, and QWK.

### Feature importance

To understand the contributions of individual input features and increase model interpretability, we utilized Shapley Additive Explanations (SHAP) values on the best performing model [52, 53]. The TreeExplainer algorithm from the SHAP Python library was used to quantify the marginal impact of each feature by averaging over all possible feature value coalitions [53]. 

## Results

### Patient population

5,904 patients met our inclusion criteria and were distributed into the early (4,686 patients with 6,186 observations); the intermediate (3,355 patients with one observation each); and the late (2,736 patients, 2,922 observations) subsets; 1,451 patients appear in all three subsets. Multiple observations per patient were possible, when patients had more than one paired ISNCSCI-SCIM assessment within the respective subset. Over 90% of the cohort sustained SCI due to traumatic causes. Most observations originated from individuals with cervical-level injuries (51%), followed by thoracic-level injuries (37%). In terms of injury severity, AIS grades A (complete injury; 38) and D (motor function preserved; 35%) were most prevalent across early, intermediate and late injury phases. Detailed demographic and injury characteristics are provided in Table 1, while Table 2 shows the class distributions for each outcome across the three subsets. The outcome distributions show clear class imbalances across all time windows. For grooming, early observations are split between class 0 (total assistance, 38%) and class 3 (independent grooming, 41%), with class 3 dominating in intermediate (63%) and late injury phases (77%). For bladder management, class 0 is most frequent early (indwelling catheter, 68%), while classes 3 (intermittent self-catheterization), and 4 (RUV < 100 cc; with or without external drainage instrument) become more predominant later. Finally, for indoor walking, class 0 dominates in early injury phases (total assistance, 58%), shifting to class 2, representing patients that moves independently in a manual wheelchair, in intermediate (51%) and late phases (46%).

**Table 1:** Demographic and clinical information of patients included in comparison to the full EMSCI cohort

Feature	Full Data Set	Subsets		
*mean (SD)*		**Early ** (< = 40 DAI) 21(11)	**Intermediate** (70–100 DAI) 83(8)	**Late** (> 182 DAI) 337(83)
**Number of patients**, *n*	5904	4686	3355	2736
**Demographics**				
**Sex**				
Male, *n (%)*	4523 (76.6%)	3588 (76.6%)	2608 (77.7%)	2121 (77.5%)
Female, *n (%)*	1381(23.4%)	1098 (23.4%)	747 (22.3%)	615 (22.5%)
**Age at injury** [years], *mean (SD)*	47.8 (19.0)	47.7 (19.0)	47.0 (19.1)	45.4 (18.3)
**Injury characteristics**				
**Cause of injury**				
Traumatic, *n (%)*	5413 (91.7%)	4277 (91.3%)	3102 (92.5%)	2539 (92.8%)
Ischemic, *n (%)*	452 (7.7%)	371 (7.9%)	226 (6.7%)	177 (6.5%)
Haemorragic, *n (%)*	12 (0.2%)	12 (0.3%)	9 (0.3%)	9 (0.3%)
Other, *n (%)*	27 (0.5%)	26 (0.5%)	18 (0.5%)	11 (0.4%)
**AIS grade** ^*^				
** A**, n (%)	6411 (37.6%)	2421 (39.1%)	1274 (38.0%)	1019 (34.9%)
** B**, n (%)	1860 (10.9%)	746 (12.1%)	337 (10.0%)	279 (9.5%)
** C**, n (%)	2584 (15.2%)	1121 (18.1%)	490 (14.6%)	290 (9.9%)
** D**, n (%)	6016 (35.3%)	1867 (30.2%)	1230 (36.7%)	1252 (42.8%)
** E**, n (%)	173 (1.0%)	31 (0.5%)	24 (0.7%)	82 (2.8%)
**Neurological level of injury**				
** Cervical**, n (%)	8675 (50.9%)	3259 (52.7%)	1680 (50.1%)	1374 (47.0%)
** Thoracic**, n (%)	6364 (37.3%)	2296 (37.1%)	1283 (38.2%)	1105 (37.8%)
** Lumbar**, n (%)	1813 (10.6%)	594 (9.6%)	364 (10.8%)	354 (12.1%)
** Sacral**, n (%)	18 (0.1%)	6 (0.1%)	3 (0.1%)	7 (0.2%)
** Intact**, n (%)	174 (1.0%)	31 (0.5%)	25 (0.7%)	82 (2.8%)

^*^American Spinal Injury Association Impairment Scale (AIS): AIS-A no sensory or motor function is preserved in the sacral segments S4-5. AIS-B sensory but no motor function is preserved below the neurological level and includes the sacral segments S4-5 (LT or PP at S4-5 or DAP), and no motor function is preserved more than three levels below the motor level on either side of the body. AIS-C motor function is preserved at the most caudal sacral segments for voluntary anal contraction OR the patient meets the criteria for sensory incomplete status, and has some sparing of motor function more than three levels below the ipsilateral motor level on either side of the body. Less than half of key muscle functions below the single NLI have a muscle grade ≥ 3. AIS-D motor incomplete status as defined above, with at least half (half or more) of key muscle functions below the single NLI having a muscle grade ≥ 3. AIS-E if sensation and motor function as tested with the ISNCSCI are graded as normal in all segments, and the patient had prior deficits, then the AIS grade is E. Someone without an initial SCI does not receive an AIS grade

Abbreviations: EMSCI, European Multicenter Study about Spinal Cord Injury; DAI, Days after injury; std, Standard deviation; AIS, American Spinal Injury Association Impairment Scale;

Only observations that are complete in all input and output features and AIS grades are presented. Percentages of sex, age, and cause of injury relate to the number of patients. Percentages of AIS grade and NLI relate to the number of observations

**Table 2:** Class distribution (n, [%]) and description of the three SCIM items used as outcome features

Spinal Cord Independence Measure (SCIM) item	Subsets		
	Early (< = 40 DAI)	Intermediate (70–100 DAI)	Late (> 182 DAI)
**Number of patients**	4686	3355	2736
**Number of observations**	6186	3355	2922
**Item 4: Grooming**			
0. Requires total assistance	2325 (38%)	604 (18%)	245 (8%)
1. Requires partial assistance	996 (16%)	415 (12%)	263 (9%)
2. Grooms independently with adaptive devices	349 (6%)	210 (6%)	162 (6%)
3. Grooms independently without adaptive devices	2516 (41%)	2126 (63%)	2252 (77%)
**Item 6: Sphincter Management - Bladder**			
0. Indwelling catheter	4217 (68%)	1091 (33%)	615 (21%)
1. RUV > 100 cc; no regular catheterization or assisted intermittent catheterization	849 (14%)	455 (14%)	177 (6%)
2. RUV < 100 cc or intermittent self-catheterization; needs assistance for applying drainage instrument	163 (3%)	168 (5%)	85 (3%)
3. Intermittent self-catheterization; does not use or not need assistance for applying external drainage instrument	321 (5%)	944 (28%)	1065 (36%)
4. RUV < 100 cc; with or without external drainage instrument	636 (10%)	697 (21%)	980 (34%)
**Item 12: Mobility - indoor walking**			
0. Requires total assistance	3600 (58%)	502 (15%)	102 (3%)
1. Electric wheelchair or partial assistance to operate a manual wheelchair	509 (8%)	353 (11%)	245 (8%)
2. Moves independently in a manual wheelchair	1464 (24%)	1715 (51%)	1345 (46%)
3. Requires supervision while walking (with or without devices)	131 (2%)	109 (3%)	64 (2%)
4. Walks with a walking frame or crutches (swing)	122 (2%)	137 (4%)	137 (5%)
5. Walks with crutches, canes, or leg orthosis only (all reciprocal walking)	96 (2%)	191 (6%)	258 (9%)
6. Walks without walking aids	264 (4%)	348 (10%)	771 (26%)

Abbreviations: DAI, Days after injury; RUV, Residual urine volume

### Model performance

**Fig. 2:**
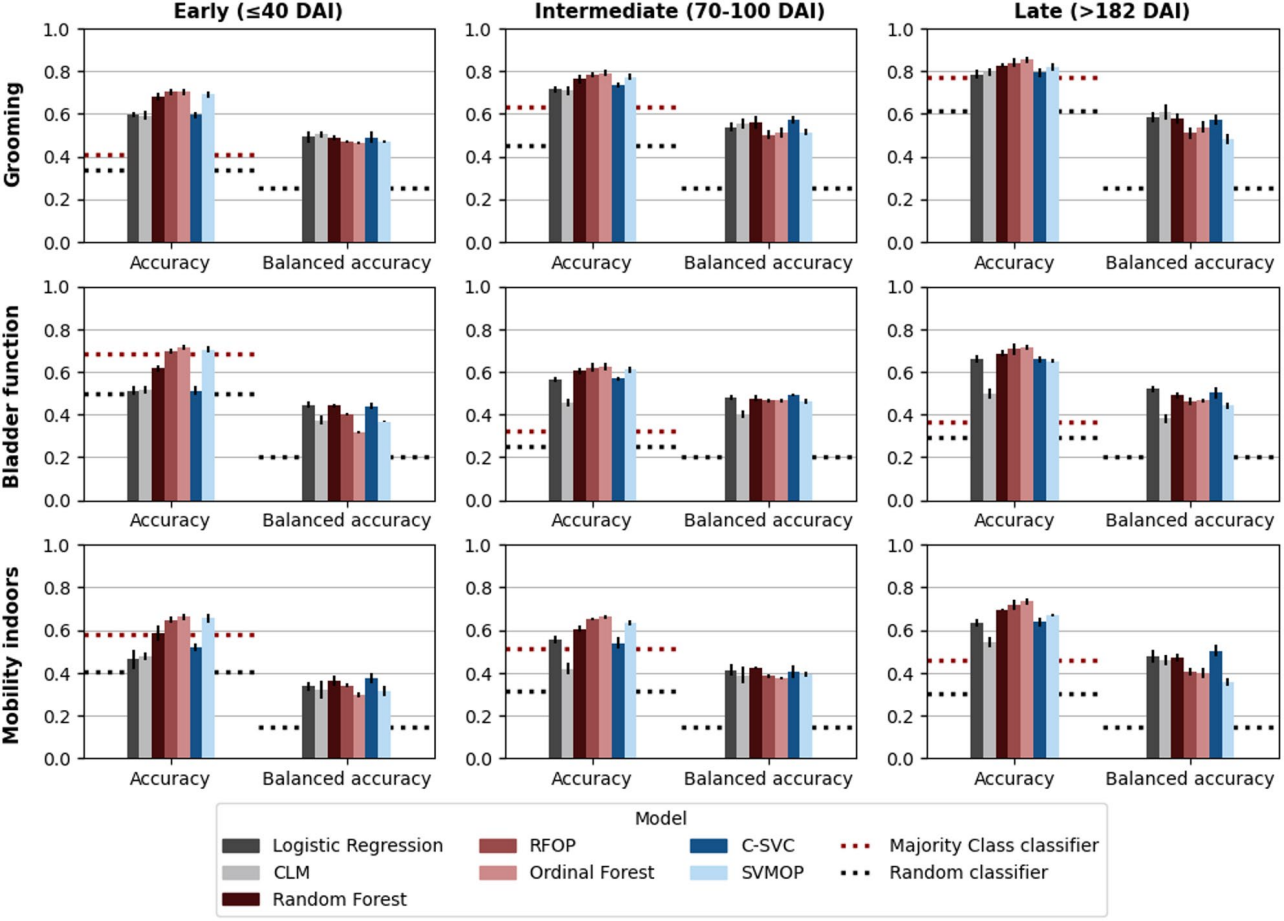
Accuracy and balanced accuracy of all predictive models evaluated across five cross-validation folds (mean ± standard deviation). Columns correspond to different data subsets representing distinct injury stages, while rows represent functional independence outcomes. Horizontal dashed lines show baseline values of naïve classifiers. The y-axis shows the performance achieved by each model. The x-axis corresponds to two evaluation methods. Each barplot corresponds to a classification model, trained on ISNCSCI assessment in input. Top row summarizes results for grooming outcome, middle row for bladder management outcome and bottom row for mobility indoors outcome. Abbreviations: DAI, Days after injury; CLM, Cumulative link model; RFOP, Random Forest with ordered partitions; C-SVC, C-Support Vector Classification; SVMOP, Support Vector Classification with ordered partitions

This section reports the predictive performance of models incorporating all individual motor and sensory scores as input features. Corresponding results for models using only sum scores are available in Tables S6 and S7. Model accuracy ranged from 0.42 to 0.85, and balanced accuracy from 0.29 to 0.61 across different model types and classification tasks (Table S4). All models outperformed random chance (ranging 0.20–0.24 when prediction task includes 4–5 possible outcomes), as illustrated in Fig. 2. Nevertheless, logistic regression, CLM and random forest performed worse than a naïve baseline classifier predicting only the majority class, in the early subset for mobility indoors and bladder function. A similar pattern was observed for the CLM in the intermediate subset when predicting independence in indoor mobility. The mean absolute error varied between 0.21 and 1.07, and macro-MAE between 0.46 and 1.56 (Table S4 and Fig. S2). QWK values ranged from 0.42 to 0.86, with higher values indicating better alignment between the predicted and actual outcome (Table S4 and Fig. S3). Across all models and classification tasks, accuracy and MAE consistently exceeded their class-balanced counterparts. This discrepancy, along with inspection of confusion matrices (Figs. S4–S10), revealed limited predictive performance for minority classes.

### Evaluation over time

For most models, classification accuracy improved over time following injury, in particular between early and late phase, while the intermediate phase showed mixed patterns. In the early subset, model performance was moderate, with accuracy values ranging from 0.59 to 0.70 for grooming, 0.51 to 0.71 for bladder function, and 0.46 to 0.66 for mobility. In the intermediate subset, accuracy increased to between 0.71 and 0.79 for grooming, while showed mixed performance for bladder function (0.45 and 0.62) and mobility indoors (0.42 and 0.66). The highest accuracies were observed in the late subset, with grooming predictions ranging from 0.79 to 0.85, bladder function from 0.50 to 0.72, and mobility from 0.55 to 0.74. Among the SCIM items, grooming yielded the most consistent and highest predictive accuracy, while bladder function and mobility exhibited more variable results across recovery phases.

When comparing ordinal and nominal variants of the same base model (Table 3), ordinal models generally achieved higher accuracy, while nominal models showed higher balanced accuracy. Moreover, ordinal models often performed better in terms of MAE, macro-MAE, and QWK. Notably, the CLM exhibited the most pronounced accuracy-QWK trade-off, ranking lowest when evaluated by accuracy and balanced accuracy (sum of ranks: 58 and 38, respectively), yet achieving the best overall QWK performance (sum of ranks: 9) as shown in Table S4. The Ordinal Forest and OP models demonstrated strong results in terms of accuracy and MAE, but performed poorly on balanced accuracy and macro-MAE, again indicating a consistent bias toward the majority classes (Figs. S7, S8, and S10). The random forest (RF) model demonstrated the most robust performance overall, achieving the highest relative performance score (0.9302 ± 0.030) and the lowest aggregated rank (31). Its predictive accuracy was stable across tasks and time points, and consistent across all evaluation metrics. The ordinal forest (0.918 ± 0.032, sum of ranks: 34) and the RFOP (0.912 ± 0.030, sum of ranks: 35) followed closely, indicating that forest-based approaches were the most reliable and effective across the evaluated scenarios.

**Table 3:** Mean and standard deviation of relative performance scores averaged over all scenarios (i.e. data subset–outcome combinations)

Model	Type	Mean (SD) over scenarios
Random Forest (RF)	Nominal	0.930 (0.030)
Ordinal Forest	Ordinal	0.918 (0.032)
Random Forest with ordered partitions (RFOP)	Ordinal	0.914 (0.030)
C-Support Vector Classification (C-SVC)	Nominal	0.911 (0.040)
Cumulative link model (CLM)	Ordinal	0.897 (0.050)
Logistic Regression	Nominal	0.895 (0.056)
Support Vector Classification with ordered partitions (SVMOP)	Ordinal	0.890 (0.056)

Relative performance was determined relative to the best model in five different metrics (accuracy, balanced accuracy, mean absolute error, macro-averaged mean absolute error and quadratic weighted kappa). All metrics were combined using a simple additive weighting approach with equal weights. Relative performance scores range from 0 to 1, where 1 indicates that the model matched the best-performing model in a given scenario, and lower values indicate proportionally lower performance

### Feature importance

We use SHAP values to quantify the contribution of input features to predictions across the three investigated SCIM items. All results shown are based on the best model, multi-class RF, and account for all ISNCSCI motor and sensory scores (including DAP and VAC); results for the reduced input are available in Fig. S17.

#### Overall feature importance patterns

Across all SCIM items and time stages, ISNCSCI MS, DAI, and age emerged as the most influential predictors (Fig. S11). DAI was especially influential in early-phase predictions but decreased in importance over time, whereas age showed increasing relevance in later recovery stages.

#### SCIM item 4: grooming

**Fig. 3:**
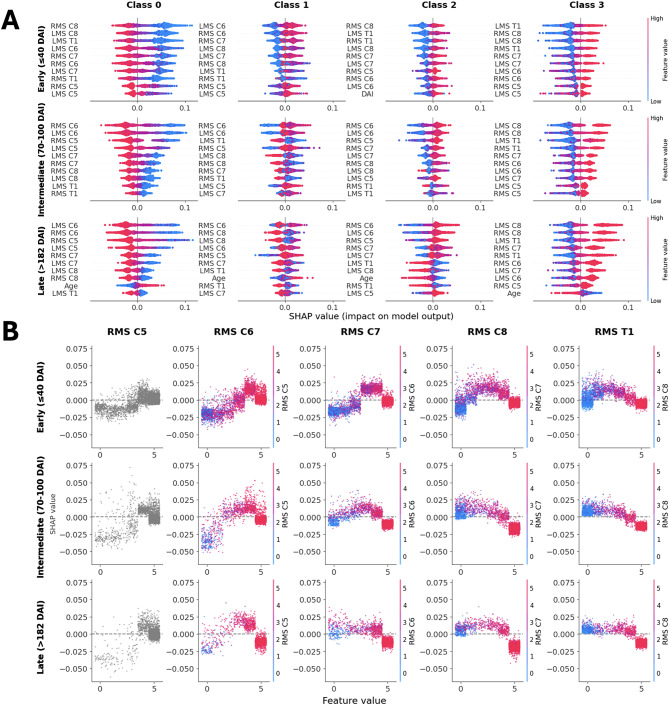
SHAP analysis of feature importance for SCIM item 4: Grooming in patients with tetraplegia. **(A)** SHAP of the ten most important features for each class. Each subplot shows the impact of the features on the model’s output for that specific outcome class (i.e., 0 requires total assistance). Features are sorted by the highest mean absolute SHAP value in descending order and data points are colored by feature value. Higher values are shown in pink, while lower values are shown in blue. Rows correspond to different data subsets representing distinct injury stages. **(B)** SHAP values of the right motor scores of the spinal levels C5 to T1 for class 1. Each subplot shows the SHAP values in relation to the feature values for that specific motor score. Data points of segments C6 to T1 are colored by the feature value of the segment that lies cranial to them. Rows correspond to different data subsets representing distinct injury stages. Abbreviations: SHAP, Shapley additive explanations; SCIM, Spinal Cord Independence Measure; DAI, Days after injury; RMS, right motor score, LMS, left motor score

Upper extremity motor scores (C5–T1) were dominant predictors of grooming ability, with C8, which innervates finger flexors, consistently emerging as the top feature. C6 MS gained importance in classes 0 to 3 during intermediate and late stages, reflecting its key role in presence of an active tenodesis grip (Figs. S14 to S16). Lower C5–T1 MS scores were linked to class 0 while intermediate scores had the strongest influence in class 1 (Fig. 3A and B). For class 2, C6 MS was positively associated with higher independence in grooming, especially in later stages (Fig. S15). Overall, motor scores of 5 were associated with class 3 predictions, and this association increased over time (Fig. S16). Age also ranked among the top predictors in the late phase, showing a positive association in lower-function classes (0,1) and a negative one in higher-function classes (2 and 3) (Fig. 3A).

#### SCIM item 6: bladder management

DAI was the most important predictor in the early phase (Fig. S12), particularly for classes 0 (positive association) and 2 and 3 (negative association). In later phases, age became more relevant, with higher age values increasing the likelihood of class 0, and decreasing class 3 predictions. VAC and lower extremity MSs were also highly predictive, especially for class 4 (full independence). A present VAC increased the likelihood of class 4 and decreased it for classes with lower independence.

#### SCIM item 12: indoor mobility

Lumbar MS, particularly L2 and L3, had the highest SHAP values across all classes, as shown in Fig. S13. These scores negatively influenced predictions of lower mobility (classes 0–2) and positively influenced higher mobility classes (3–6). In later stages, L4 and L5 MS showed a similar trend, while S1 MS was among the top features across all phases. Notably, C6, C8, and T1 MS gained relevance in later phases, particularly for all classes except class 5. Age and DAI were again influential, with DAI primarily affecting early-stage predictions and age showing class-specific associations: positive in class 0 and negative in classes 2 and 5. Older age showed a positive association in class 3 (supervision during walking).

## Discussion

In this study, we systematically evaluated a suite of machine learning models to classify SCIM-based functional independence outcomes in individuals with SCI utilizing data from EMSCI. Our comprehensive assessment of cross-sectional relationships between neurological status and functional independence extends previous investigations of longitudinal neurological-functional relationships in SCI populations [5, 6, 10]. We show that functional independence in specific tasks can be predicted from concurrent neurological assessment results, using both multi-class and ordinal classification models.

Across three clinical domains of functional independence, namely grooming, bladder management, and indoor mobility, forest-based algorithms consistently outperformed other machine learning approaches. RF, Ordinal Forest, and RFOP achieved higher accuracy at early, intermediate and late time points with RF performing best overall across all classification scenarios.

### Temporal dynamic of feature importance

Model performance generally improved for later stages after injury peaking in the late phase (> 182 days post-injury), which indicates a more stable association between neurological assessment results and functional independence. This progression likely reflects confounding factors present early after traumatic SCI [54]. Functional outcome classification early after SCI, aside from the high class imbalance, presents particular challenges due to ongoing neurological changes and the presence of e.g. concomitant injuries or occurrence of secondary complications [55].

Additionally, the temporal evolution of feature importance provides insights into recovery mechanisms. For grooming tasks, the increasing importance of C6 motor scores over time likely reflects both neurological recovery and learned compensatory strategies, particularly the development of tenodesis grasp patterns through rehabilitation [39]. This finding suggests that the models may capture both static neurological capacity and functional adaptations.

### Ordinal nature of outcome targets

We showed that ordinal models did not systematically outperform nominal models suggesting that SCIM item categories, though ordinal by design, may not encode ordinal progressions in functional independence. However, ordinal models performed better in evaluation metrics that penalize the severity of misclassifications, suggesting that such models make fewer severe misclassifications. This property is highly relevant for applications in SCI care, where the magnitude of functional changes (e.g., walking unaided vs. wheelchair-dependent mobility) has profound implications for patient management. Therefore, ordinal models may be preferred in clinical settings where the cost of severe misclassification is high, as different levels of functional independence are associated with substantially different assistance requirements and clinical management [42]. 

### Neurological - functional independence associations

Interpretability analyses using SHAP provided insights into model decision-making and confirmed their clinical plausibility. For grooming, upper extremity motor scores at cervical spinal levels C6–C8 emerged as dominant predictors, with C6 (wrist extension) showing particular importance as it potentially enables a tenodesis grasp [5, 31]. Motor scores of lumbar myotomes dominated predictions of indoor mobility, with L2 (hip flexors) and L3 (knee extensors) showing greatest influence, aligning with established knowledge [6, 7, 18, 28, 56, 57]. Raw SHAP analysis revealed that L2 and L3 scores primarily distinguished walkers from non-walkers, while L4 and L5 scores (ankle dorsiflexors and toe extensors) differentiated walking aid requirements among ambulatory patients, supporting the clinical understanding that proximal muscle strength enables walking capacity, while distal strength determines walking quality and independence [7].

Upper extremity scores (C6, C8, T1) gained importance for classes representing the use of a self-propelled wheelchair and walking aids in intermediate and late phases, reflecting their role in enabling assistive device use. Our results demonstrate that levels of functional independence can be classified from neurological assessment results. SHAP analysis confirms that classification models are relying on anatomically sound relationships between spinal segments and corresponding functional capacities. These results provide an initial step towards a complete, and validated data-driven framework for understanding how neurological status relates to functional independence.

### Limitations

Model performance was evaluated using five-fold cross-validation, a widely accepted method for internal validation, however, this approach may overestimate generalisability. External validation on a different dataset would be required to provide a robust assessment of model generalisability prior to clinical application.

Class imbalance may have further biased model predictions. Although class weights and suitable evaluation metrics were applied, models remained biased toward majority classes, as reflected by reduced performance for minority class. Resampling techniques, such as SMOTE [58], to balance class distributions, may further improve results. Moreover, while ISNCSCI provides standardized assessment, its limitation to 10 key muscles per body side may not capture the complete neurological status of a patient. Integration with additional assessments such as electrophysiological measures may enhance predictive ability. This study focused on neurological and demographic predictors, but functional independence outcomes are influenced by other variables including psychological factors, social support, confounding factors, all information not available in EMSCI. Integrating these factors would likely improve predictive accuracy and in particular clinical utility. Additionally, we predict one task for each of the three domains of the SCIM, aligning with previous literature. However, these tasks do not fully capture all aspects of functional independence for people living with SCI. Finally, although providing valuable insights, the interpretation of SHAP values should be approached with caution, as these values do not imply causality. Given the limited performance for minority class predictions, feature importance analysis should be approached particularly carefully for these cases. Furthermore, potential correlations and interactions between features, factors known to influence SHAP values and complicate their interpretation, were not explored in this study.

## Conclusion

In this work, we show that data-driven models can reliably predict functional independence measured by three different SCIM items from concurrent neurological assessments in different post-injury phases over the first year after the injury. The improvement in classification performance for later stages after SCI indicates that model reliability increases as the neurological recovery plateaus, providing clinicians with increasingly confident models through the recovery trajectory. Random forest algorithms achieved the best overall performance, while ordinal classification models can offer particular clinical value by reducing the risk of severe misclassifications, a crucial advantage in medical applications. Feature importance analysis revealed changes over time reflecting both neurological recovery and the potential impact of rehabilitation, suggesting that models are able to capture clinically relevant recovery dynamics. Importantly, the classification models developed in this study can be implemented within a modular framework in combination with models that predict future motor and sensory scores ultimately aiming for functional recovery: first future neurological status (e.g., motor scores at 6 months post-injury) is predicted, followed by functional independence classification from predicted neurological scores based on the presented models developed in this study. This modular setup eliminates the need to develop separate models for every combination of functional task and time point after injury, creating a computationally efficient and scalable approach to prognostic modeling in SCI care.

In conclusion, the integration of interpretable machine learning with standardized clinical assessments represents a promising approach for optimizing SCI care and improving patient outcomes.

## Supplementary Information

Below is the link to the electronic supplementary material.

Supplementary Material 1

## Data Availability

The data used for this study, including de-identified individual participant data and a data dictionary defining each field or variable within the dataset, can be made available on reasonable request to the corresponding author (CRJ). These data will be made available following publication of this work. Written proposals will be evaluated by the authors, who will render a decision regarding suitability and appropriateness of the use of data. Approval of all authors will be required and a data sharing agreement must be signed before any data are shared. The code to run the analysis as well as create the figures and tables can be found at our Gitlab repository: https://gitlab.ethz.ch/BMDSlab/publications/sci/neuro\_to\_functional.

## Abbreviations

SCI: Spinal cord injury
DAP: Deep anal pressure
VAC: Voluntary anal contraction
SCIM: Spinal cord independence measure
SHAP: Shapley additive explanations
ISNCSCI: International standards for neurological classification of spinal cord injury
EMSCI: European multicenter study about spinal cord injury
MS: Motor score
AIS: American spinal injury association impairment scale
ASIA: American spinal injury association
STROBE: Strengthening the reporting of observational studies in epidemiology
DAI: Days after injury
SS: Sensory score
UEMS: Upper extremity motor score
LEMS: Lower extremity motor score
RUEMS: Right upper extremity motor score
LUEMS: Left upper extremity motor score
RLEMS: Right lower extremity motor score
LLEMS: Left lower extremity motor score
RPP: Right pinprick
LPP: Left pinprick
RLT: Right light touch
LLT: Left light touch
SVM: Support vector machine
CLM: Cumulative link model
OP: Ordered partitions
MAE: Mean absolute error
QWK: Quadratic weighted kappa
RF: Random forest
RFOP: Random forest with ordered partitions
