# Supplementary material for: Linking neurological status to functional outcomes in spinal cord injury: a multi-class, task-specific approach
Source: BMC Biomed Eng. 2026 Apr 15;8:9. doi: 10.1186/s42490-026-00110-1 (PMC13154549; doi:10.1186/s42490-026-00110-1)
Supplement: Supplementary file 1 — Supplementary Material 1 [file 42490_2026_110_MOESM1_ESM.docx]

# **Supplementary material**

## Supplementary methods

### Mapping SCIM Versions

**Table S1:** Mappings to harmonize SCIM version II and III for items 4 and 6. Numbers represent the original scores assigned to the classes. SCIM, Spinal Cord Independence Measure.

| **SCIM II** | | **SCIM III** | |
| --- | --- | --- | --- |
| **SCIM item 4: Grooming** | **0**  **1**  **2**  **3**  **4** | **SCIM item 4: Grooming** | **0**  **1**  **1**  **2**  **3** |
|  |  |  |  |
|  |  |  |  |
|  |  |  |  |
|  |  |  |  |
| **SCIM III** | | **SCIM II** | |
| **SCIM item 6: Sphincter Management - Bladder** | **0**  **3**  **6**  **9**  **11**  **13**  **15** | **SCIM item 6: Sphincter Management - Bladder** | **0**  **4**  **8**  **12**  **12**  **15**  **15** |
|  |  |  |  |
|  |  |  |  |
|  |  |  |  |
|  |  |  |  |

### Merging subscores for individual SCIM items

**Table S2:** Subscores for SCIM subitems 6 and 12 were merged to increase the number of samples available per score and improve model stability. RUV: residual urine volume.

| **SCIM item 6: sphincter management - bladder** | | **SCIM item 12: mobility - indoor walking** | |
| --- | --- | --- | --- |
| **initial subscore** | **merged subscore** | **initial subscore** | **merged subscore** |
| 0 (indwelling catheter) | 0 | 0 (requires total assistance) | 0 |
| 3 (RUV > 100cc; no regular catheterization or assisted intermittent catheterization) | 1 | 1 (needs electric wheelchair or partial assistance to operate manual wheelchair) | 1 |
| 6 ( RUV < 100cc or intermittent self-catheterization; needs assistance for applying drainage instrument) | 2 | 2 (moves independently in manual wheelchair) | 2 |
| 9 (Intermittent self-catheterization; uses external drainage instrument; no need for assistance) | 3 | 3 (requires supervision while walking, with or without devices) | 3 |
| 11 (Intermittent self-catheterization; continent between catheterizations; no need for external drainage instrument) | 3 | 4 (walks with a walking frame or crutches, swing) | 4 |
| 13 (RUV < 100cc; needs only external urine drainage; no assistance required) | 4 | 5 (walks with crutches or two canes, reciprocal) | 5 |
| 15 (RUV < 100cc; continent; does not use external drainage instrument) | 4 | 6 (walks with one cane) | 5 |
|  |  | 7 (needs leg orthosis only | 5 |
|  |  | 8 (walks without walking aids) | 6 |

### For SCIM item 6, subscores 9 and 11 were merged as they represent the ability to independently self-catheterize, and subscores 13 and 15 were merged as they represent low residual volume and no need for intermittent self-catheterization. For SCIM item 12, subscores 5, 6 and 7 were merged as they all represent independent reciprocal walking with varying need for assistive devices.

### Hyperparameters evaluated

**Table S3:** Hyperparameter evaluated. CLM, Cumulative link model; RF, Random Forest; RFOP, Random Forest with ordered partitions; C-SVC, C-Support Vector Classification; SVMOP, Support Vector Classification with ordered partitions.

| **Link function models** | | | | | | |
| --- | --- | --- | --- | --- | --- | --- |
|  | Solver | Max Iterations | L2-Regularization | Link functions | | |
| **Logistic Regression** | saga | 5000 | 0,0.01, 1, 10, 100 | logit | | |
| **CLM** | Coordinate descent | 100 | 0, 0.001, 0.01, 0.1, 1 | Logit, probit, cloglog, cauchit | | |
| **Forest-based models** | | | | | | |
|  | Number of estimators | Min samples at leafs node | Max Features at Split | Min samples at split | Max  depth | Bootstrap |
| **RF and RFOP** | 100, 200, 500 | 1 | Sqrt, log2 | 2, 5, 10 | None, 10, 20 | True |
|  | Number of trees in final forest | Min samples at leaf node | Max features at split | Number of sets | Number of trees per subset | Performance function |
| **Ordinal Forest** | 5000 | 1, 5 | Sqrt, 2*sqrt | 1000 | 100 | equal |
| **SVC-based models** | | | | | | |
|  | C | Kernel | Gamma | Degree of poly kernel | | |
| **C-SVC** | 0.01, 0.1, 1, 10, 100 | Linear, poly, rbf, sigmoid | Scale, auto | 2, 3, 4 | | |
| **SVMOP** | 0.01, 0.1, 1, 10 | rbf | 0.01, 0.1, 1, 10 | NA | | |

###

##

## Supplementary results

### Subset selection


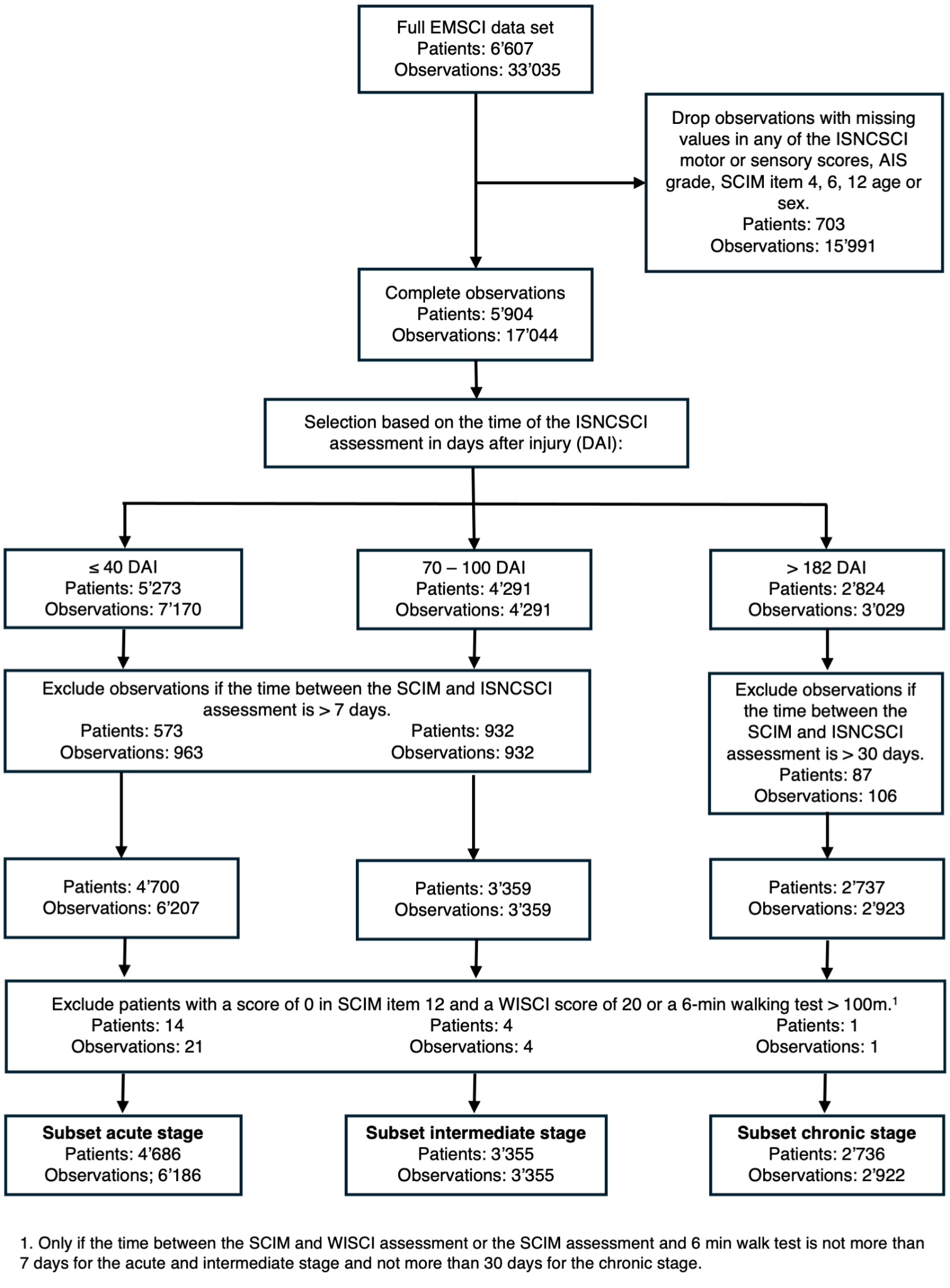


**Figure S1.** Subset selection. EMSCI, European Multicenter Study about Spinal Cord Injury; ISNCSCI, International Standards for the Neurological Classification of Spinal Cord Injury; AIS, American Spinal Injury Association impairment scale; SCIM, Spinal Cord Independence Measure; WISCI, Walking Index for Spinal Cord Injury.

##

## Supplementary results for complete input version

In the following, supplementary results of the model versions using all ISNCSCI MS and SS as input features are shown.

### Model performance

**Table S4:** Sum of ranks aggregated over all scenarios (i.e. data subset–outcome combinations). In each scenario, rank 1 represents the best performance; depending on the metric, the best performance might correspond to the highest or lowest score. As the total number of scenarios (see *Classification Models*) is nine, the lowest possible number of ranks equals nine. The best model within each metric is marked in bold.

|  | Accuracy | Balanced Accuracy | Mean Accuracy | MAE | Macro-MAE | Mean MAE | QWK | Mean all |
| --- | --- | --- | --- | --- | --- | --- | --- | --- |
| Random Forest | 33 | 23 | **28.0** | 31 | 34 | **32.5** | 35 | **31.0** |
| Ordinal Forest | **9** | 54 | 31.5 | **13** | 56 | 34.5 | 36 | 34.0 |
| CLM | 58 | 38 | 48.0 | 52 | **15** | 33.5 | **9** | 34.0 |
| RFOP | 20 | 46 | 33.0 | 21 | 53 | 37.0 | 33 | 35.0 |
| C-SVC | 50 | **19** | 34.5 | 49 | 20 | 34.5 | 46 | 37.0 |
| Logistic Regression | 52 | 20 | 36.0 | 53 | 21 | 37.0 | 49 | 39.0 |
| SVMOP | 33 | 52 | 41.0 | 33 | 53 | 43.0 | 44 | 42.0 |

*Abbreviations: MAE, mean absolute error, Macro-MAE, macro-averaged mean absolute error; QWK, quadratic weighted kappa; RFOP, Random Forest with ordered partitions; CLM, Cumulative link model; C-SVC, C-Support Vector Classification; SVMOP, Support Vector Classification with ordered partitions; CNN, Convolutional Neural Network.*

**Table S5**: Results of all models in five different evaluation metrics. Mean and standard deviation over the five cross-validation folds are displayed. The best model(s) within each metric is marked in bold. MAE, mean absolute error, Macro-MAE, macro-averaged mean absolute error; QWK, quadratic weighted kappa; DAI, Days after injury; CLM, Cumulative link model; RFOP, Random Forest with ordered partitions; C-SVC, C-Support Vector Classification; SVMOP, Support Vector Classification with ordered partitions; CNN, Convolutional Neural Network.

|  | Grooming | Bladder | Mobility indoors | Grooming | Bladder | Mobility indoors | Grooming | Bladder | Mobility indoors | Grooming | Bladder | Mobility indoors | Grooming | Bladder | Mobility indoors |
| --- | --- | --- | --- | --- | --- | --- | --- | --- | --- | --- | --- | --- | --- | --- | --- |
|  | **Accuracy** | | | **Balanced Accuracy** | | | **MAE** | | | **Macro-MAE** | | | **QWK** | | |
| **Acute stage (≤40 DAI)** | | | | | | | | | | | | | | | |
| Logistic Regression | 0.60 (0.01) | 0.51 (0.02) | 0.46 (0.05) | 0.49 (0.03) | 0.45 (0.02) | 0.34 (0.02) | 0.58 (0.04) | 1.07 (0.08) | 1.04 (0.09) | 0.67 (0.06) | 0.97 (0.05) | 1.22 (0.10) | 0.71 (0.03) | 0.42 (0.02) | 0.53 (0.07) |
| CLM | 0.59 (0.02) | 0.51 (0.02) | 0.48 (0.02) | 0.50 (0.02) | 0.37 (0.02) | 0.32 (0.04) | 0.55 (0.03) | 0.87 (0.03) | 0.89 (0.02) | 0.62 (0.02) | 0.98 (0.03) | 1.08 (0.08) | 0.74 (0.02) | 0.52 (0.04) | 0.64 (0.02) |
| Random Forest | 0.68 (0.02) | 0.62 (0.02) | 0.58 (0.03) | 0.49 (0.01) | 0.44 (0.01) | 0.36 (0.02) | 0.53 (0.03) | 0.91 (0.04) | 0.86 (0.05) | 0.73 (0.03) | 1.05 (0.02) | 1.17 (0.06) | 0.72 (0.02) | 0.46 (0.02) | 0.61 (0.01) |
| Ordinal Forest | 0.70 (0.01) | 0.71 (0.01) | 0.66 (0.01) | 0.46 (0.01) | 0.32 (0.01) | 0.29 (0.01) | 0.54 (0.04) | 0.61 (0.03) | 0.69 (0.03) | 0.80 (0.02) | 1.50 (0.03) | 1.56 (0.05) | 0.71 (0.03) | 0.48 (0.02) | 0.62 (0.03) |
| RFOP | 0.70 (0.01) | 0.70 (0.01) | 0.65 (0.02) | 0.47 (0.01) | 0.40 (0.01) | 0.34 (0.01) | 0.54 (0.03) | 0.72 (0.04) | 0.80 (0.03) | 0.79 (0.02) | 1.23 (0.03) | 1.39 (0.04) | 0.71 (0.02) | 0.51 (0.02) | 0.62 (0.02) |
| C-SVC | 0.59 (0.02) | 0.51 (0.02) | 0.52 (0.02) | 0.49 (0.03) | 0.44 (0.01) | 0.37 (0.02) | 0.58 (0.04) | 1.05 (0.03) | 0.88 (0.03) | 0.68 (0.06) | 0.97 (0.03) | 1.09 (0.04) | 0.71 (0.03) | 0.42 (0.03) | 0.61 (0.02) |
| SVMOP | 0.69 (0.01) | 0.70 (0.01) | 0.66 (0.02) | 0.47 (0.01) | 0.37 (0.00) | 0.31 (0.02) | 0.54 (0.04) | 0.65 (0.04) | 0.70 (0.04) | 0.77 (0.02) | 1.33 (0.02) | 1.51 (0.09) | 0.71 (0.03) | 0.49 (0.03) | 0.62 (0.03) |
| CNN | 0.66 (0.02) | 0.69 (0.03) | 0.64 (0.01) | 0.46 (0.01) | 0.31 (0.02) | 0.30 (0.02) | 0.61 (0.03) | 0.67 (0.09) | 0.75 (0.02) | 0.84 (0.05) | 1.47 (0.12) | 1.50 (0.06) | 0.66 (0.03) | 0.47 (0.09) | 0.65 (0.04) |
| **Intermediate stage (70 - 100 DAI)** | | | | | | | | | | | | | | | |
| Logistic Regression | 0.72 (0.02) | 0.56 (0.01) | 0.55 (0.02) | 0.54 (0.02) | 0.48 (0.01) | 0.41 (0.02) | 0.38 (0.02) | 0.82 (0.05) | 0.73 (0.04) | 0.58 (0.04) | 0.88 (0.07) | 0.93 (0.04) | 0.79 (0.02) | 0.59 (0.05) | 0.76 (0.02) |
| CLM | 0.71 (0.02) | 0.45 (0.02) | 0.42 (0.02) | 0.55 (0.03) | 0.40 (0.02) | 0.39 (0.04) | 0.36 (0.03) | 0.85 (0.03) | 0.79 (0.05) | 0.53 (0.04)} | 0.90 (0.04) | 0.86 (0.07)} | 0.81 (0.02) | 0.64 (0.02) | 0.78 (0.02) |
| Random Forest | 0.76 (0.02) | 0.60 (0.01) | 0.61 (0.01) | 0.56 (0.03) | 0.48 (0.01) | 0.42 (0.01) | 0.34 (0.04) | 0.80 (0.03) | 0.69 (0.03) | 0.57 (0.05) | 0.93 (0.01) | 0.94 (0.03) | 0.80 (0.03) | 0.60 (0.02) | 0.76 (0.02) |
| Ordinal Forest | 0.79 (0.02)} | 0.62 (0.02) | 0.66 (0.01) | 0.51 (0.02) | 0.46 (0.01) | 0.37 (0.01) | 0.33 (0.04) | 0.77 (0.02) | 0.63 (0.03) | 0.68 (0.05) | 0.96 (0.02) | 1.09 (0.03) | 0.79 (0.03) | 0.63 (0.01) | 0.76 (0.03) |
| C-SVC | 0.74 (0.01) | 0.57 (0.01) | 0.54 (0.03) | 0.57 (0.02) | 0.49 (0.01) | 0.41 (0.03) | 0.36 (0.03) | 0.83 (0.04) | 0.72 (0.03) | 0.54 (0.03) | 0.87 (0.03) | 0.93 (0.03) | 0.79 (0.02) | 0.58 (0.03) | 0.77 (0.02) |
| SVMOP | 0.77 (0.02) | 0.61 (0.02) | 0.63 (0.01) | 0.51 (0.01) | 0.46 (0.01) | 0.39 (0.01) | 0.34 (0.04) | 0.76 (0.04) | 0.65 (0.03) | 0.66 (0.04) | 0.93 (0.03) | 1.03 (0.02) | 0.79 (0.03) | 0.64 (0.02) | 0.76 (0.02) |
| CNN | 0.75 (0.03) | 0.60 (0.03) | 0.64 (0.01) | 0.49 (0.05) | 0.46 (0.03) | 0.37 (0.04) | 0.40 (0.07) | 0.84 (0.08) | 0.69 (0.03) | 0.74 (0.09) | 1.00 (0.11) | 1.14 (0.08) | 0.74 (0.07) | 0.59 (0.06) | 0.72 (0.04) |
| **late stage (>182 DAI)** | | | | | | | | | | | | | | | |
| Logistic Regression | 0.79 (0.02) | 0.66 (0.02) | 0.63 (0.02) | 0.58 (0.02) | 0.52 (0.02) | 0.48 (0.03) | 0.30 (0.04) | 0.63 (0.05) | 0.59 (0.03) | 0.53 (0.04) | 0.82 (0.03) | 0.82 (0.08) | 0.75 (0.05) | 0.63 (0.04) | 0.84 (0.01) |
| CLM | 0.80 (0.02) | 0.50 (0.02) | 0.55 (0.03) | 0.61 (0.04) | 0.38 (0.02) | 0.46 (0.02) | 0.24 (0.02) | 0.72 (0.02) | 0.63 (0.02) | 0.46 (0.03) | 0.90 (0.03) | 0.75 (0.07) | 0.82 (0.03) | 0.68 (0.02) | 0.86 (0.01) |
| Random Forest | 0.83 (0.01) | 0.69 (0.01) | 0.70 (0.00) | 0.58 (0.03) | 0.49 (0.02) | 0.47 (0.02) | 0.24 (0.01) | 0.64 (0.05) | 0.52 (0.02) | 0.53 (0.03) | 0.94 (0.05) | 0.84 (0.03) | 0.79 (0.04) | 0.62 (0.04) | 0.85 (0.01) |
| Ordinal Forest | 0.85 (0.01)} | 0.71 (0.01) | 0.74 (0.01) | 0.54 (0.03) | 0.47 (0.01) | 0.40 (0.02) | 0.21 (0.03) | 0.59 (0.01) | 0.49 (0.02) | 0.62 (0.05) | 0.97 (0.02) | 1.00 (0.04) | 0.80 (0.04) | 0.66 (0.02) | 0.84 (0.01) |
| RFOP | 0.84 (0.02) | 0.71 (0.03) | 0.72 (0.03) | 0.51 (0.03) | 0.46 (0.02) | 0.40 (0.02) | 0.24 (0.03) | 0.60 (0.05) | 0.52 (0.03) | 0.68 (0.05) | 0.97 (0.04) | 1.03 (0.07) | 0.78 (0.05) | 0.66 (0.05) | 0.84 (0.01) |
| C-SVC | 0.79 (0.02) | 0.66 (0.01) | 0.64 (0.02) | 0.57 (0.02) | 0.50 (0.03) | 0.50 (0.03) | 0.28 (0.02) | 0.65 (0.05) | 0.59 (0.03) | 0.53 (0.04) | 0.86 (0.07) | 0.78 (0.09) | 0.77 (0.03) | 0.62 (0.04) | 0.84 (0.01) |
| SVMOP | 0.82 (0.02) | 0.65 (0.01) | 0.67 (0.01) | 0.48 (0.02) | 0.44 (0.01) | 0.36 (0.02) | 0.28 (0.03) | 0.69 (0.04) | 0.61 (0.02) | 0.74 (0.06) | 1.00 (0.07) | 1.07 (0.10) | 0.71 (0.05) | 0.61 (0.03) | 0.80 (0.01) |
| CNN | 0.81 (0.03) | 0.72 (0.04) | 0.70 (0.01) | 0.53 (0.10) | 0.49 (0.04) | 0.40 (0.01) | 0.29 (0.03) | 0.55 (0.05) | 0.54 (0.03) | 0.63 (0.14) | 0.89 (0.09) | 1.01 (0.08) | 0.72 (0.05) | 0.68 (0.04) | 0.83 (0.01) |

**Table S6:** Results of all ordinal model versions in five different evaluation metrics. Mean and standard deviation over the five cross-validation folds are displayed. In each version of the model, the evaluation metric used for hyperparameter optimisation is shown in brackets behind the model name: BA = balanced accuracy, MAE = macro-averaged MAE, QWK. DAI, Days after injury; QWK, quadratic weighted kappa; MAE, mean absolute error, Macro-MAE, macro-averaged mean absolute error; RFOP, Random Forest with ordered partitions; C-SVC, C-Support Vector Classification; CLM, Cumulative link model; SVMOP, Support Vector Classification with ordered partitions.

|  | Grooming | Bladder function | Mobility indoors | Grooming | Bladder function | Mobility indoors | Grooming | Bladder function | Mobility indoors | Grooming | Bladder function | Mobility indoors | Grooming | Bladder function | Mobility indoors |
| --- | --- | --- | --- | --- | --- | --- | --- | --- | --- | --- | --- | --- | --- | --- | --- |
|  | Accuracy | | | Balanced Accuracy | | | MAE | | | Macro Averaged MAE | | | QWK | | |
| **Acute stage (≤40 DAI)** | | | |  | | |  | | |  | | |  | | |
| CLM (BA) | 0.57 (0.01) | 0.43 (0.03) | 0.39 (0.06) | 0.50 (0.01) | 0.38 (0.02) | 0.31 (0.03) | 0.56 (0.01) | 1.08 (0.05) | 1.00 (0.09) | 0.62 (0.02) | 0.98 (0.05) | 1.09 (0.06) | 0.74 (0.01) | 0.44 (0.04) | 0.61 (0.03) |
| CLM (MAE) | 0.54 (0.02) | 0.34 (0.02) | 0.33 (0.01) | 0.50 (0.01) | 0.34 (0.02) | 0.31 (0.04) | 0.58 (0.03) | 1.05 (0.03) | 1.01 (0.03) | 0.60 (0.02) | 0.92 (0.03) | 0.99 (0.07) | 0.73 (0.02) | 0.47 (0.03) | 0.60 (0.02) |
| CLM (QWK) | 0.59 (0.02) | 0.51 (0.02) | 0.48 (0.02) | 0.50 (0.02) | 0.37 (0.02) | 0.32 (0.04) | 0.55 (0.03) | 0.87 (0.03) | 0.89 (0.02) | 0.62 (0.02) | 0.98 (0.03) | 1.08 (0.08) | 0.74 (0.02) | 0.52 (0.04) | 0.64 (0.02) |
| Ordinal Forest (BA) | 0.70 (0.02) | 0.72 (0.01) | 0.66 (0.01) | 0.46 (0.01) | 0.31 (0.01) | 0.30 (0.01) | 0.54 (0.04) | 0.62 (0.03) | 0.69 (0.02) | 0.80 (0.02) | 1.52 (0.05) | 1.56 (0.04) | 0.71 (0.03) | 0.47 (0.02) | 0.62 (0.03) |
| Ordinal Forest (MAE) | 0.70 (0.01) | 0.71 (0.01) | 0.66 (0.01) | 0.46 (0.01) | 0.32 (0.01) | 0.29 (0.01) | 0.54 (0.04) | 0.61 (0.03) | 0.69 (0.03) | 0.80 (0.02) | 1.50 (0.03) | 1.56 (0.05) | 0.71 (0.03) | 0.48 (0.02) | 0.62 (0.03) |
| Ordinal Forest (QWK) | 0.70 (0.02) | 0.71 (0.01) | 0.66 (0.01) | 0.46 (0.01) | 0.32 (0.01) | 0.29 (0.01) | 0.54 (0.04) | 0.61 (0.03) | 0.69 (0.02) | 0.81 (0.02) | 1.50 (0.03) | 1.55 (0.04) | 0.71 (0.03) | 0.48 (0.01) | 0.62 (0.03) |
| RFOP (BA) | 0.70 (0.01) | 0.70 (0.01) | 0.65 (0.02) | 0.47 (0.01) | 0.40 (0.01) | 0.33 (0.01) | 0.54 (0.03) | 0.72 (0.03) | 0.80 (0.03) | 0.80 (0.02) | 1.23 (0.03) | 1.41 (0.03) | 0.71 (0.02) | 0.51 (0.02) | 0.62 (0.02) |
| RFOP (MAE) | 0.70 (0.01) | 0.70 (0.01) | 0.65 (0.02) | 0.47 (0.01) | 0.40 (0.01) | 0.34 (0.01) | 0.54 (0.03) | 0.72 (0.04) | 0.80 (0.03) | 0.79 (0.02) | 1.23 (0.03) | 1.39 (0.04) | 0.71 (0.02) | 0.51 (0.02) | 0.62 (0.02) |
| RFOP (QWK) | 0.70 (0.02) | 0.71 (0.01) | 0.66 (0.01) | 0.46 (0.01) | 0.38 (0.03) | 0.32 (0.01) | 0.55 (0.04) | 0.68 (0.04) | 0.73 (0.03) | 0.81 (0.03) | 1.31 (0.08) | 1.50 (0.04) | 0.70 (0.03) | 0.50 (0.03) | 0.63 (0.01) |
| SVMOP (BA) | 0.69 (0.01) | 0.70 (0.01) | 0.65 (0.02) | 0.47 (0.01) | 0.37 (0.00) | 0.31 (0.02) | 0.54 (0.04) | 0.65 (0.04) | 0.70 (0.04) | 0.77 (0.02) | 1.33 (0.02) | 1.52 (0.08) | 0.71 (0.03) | 0.49 (0.03) | 0.62 (0.03) |
| SVMOP (MAE) | 0.69 (0.01) | 0.70 (0.01) | 0.66 (0.02) | 0.47 (0.01) | 0.37 (0.00) | 0.31 (0.02) | 0.54 (0.04) | 0.65 (0.04) | 0.70 (0.04) | 0.77 (0.02) | 1.33 (0.02) | 1.51 (0.09) | 0.71 (0.03) | 0.49 (0.03) | 0.62 (0.03) |
| SVMOP (QWK) | 0.69 (0.01) | 0.70 (0.01) | 0.66 (0.02) | 0.47 (0.01) | 0.37 (0.00) | 0.31 (0.02) | 0.54 (0.04) | 0.65 (0.04) | 0.70 (0.04) | 0.77 (0.02) | 1.33 (0.02) | 1.51 (0.09) | 0.71 (0.03) | 0.49 (0.03) | 0.62 (0.03) |
| **Intermediate stage (70-100 DAI)** | | | |  |  |  |  |  |  |  |  |  |  |  |  |
| CLM (BA) | 0.71 (0.02) | 0.45 (0.01) | 0.46 (0.01) | 0.55 (0.03) | 0.41 (0.02) | 0.40 (0.03) | 0.37 (0.03) | 0.88 (0.01) | 0.77 (0.05) | 0.54 (0.03) | 0.90 (0.02) | 0.86 (0.07) | 0.81 (0.02) | 0.62 (0.01) | 0.77 (0.02) |
| CLM (MAE) | 0.69 (0.03) | 0.39 (0.01) | 0.46 (0.01) | 0.56 (0.02) | 0.40 (0.01) | 0.39 (0.04) | 0.38 (0.03) | 0.89 (0.02) | 0.75 (0.04) | 0.52 (0.03) | 0.84 (0.02) | 0.83 (0.06) | 0.81 (0.02) | 0.61 (0.01) | 0.78 (0.02) |
| CLM (QWK) | 0.71 (0.02) | 0.45 (0.02) | 0.42 (0.02) | 0.55 (0.03) | 0.40 (0.02) | 0.39 (0.04) | 0.36 (0.03) | 0.85 (0.03) | 0.79 (0.05) | 0.53 (0.04) | 0.90 (0.04) | 0.86 (0.07) | 0.81 (0.02) | 0.64 (0.02) | 0.78 (0.02) |
| Ordinal Forest (BA) | 0.79 (0.01) | 0.63 (0.02) | 0.66 (0.01) | 0.51 (0.02) | 0.47 (0.01) | 0.37 (0.01) | 0.33 (0.03) | 0.77 (0.03) | 0.63 (0.03) | 0.68 (0.04) | 0.95 (0.01) | 1.09 (0.05) | 0.79 (0.03) | 0.63 (0.02) | 0.75 (0.02) |
| Ordinal Forest (MAE) | 0.79 (0.02) | 0.62 (0.02) | 0.66 (0.01) | 0.51 (0.02) | 0.46 (0.01) | 0.37 (0.01) | 0.33 (0.04) | 0.77 (0.02) | 0.63 (0.03) | 0.68 (0.05) | 0.96 (0.02) | 1.09 (0.03) | 0.79 (0.03) | 0.63 (0.01) | 0.76 (0.03) |
| Ordinal Forest (QWK) | 0.79 (0.02) | 0.63 (0.02) | 0.66 (0.01) | 0.51 (0.02) | 0.46 (0.01) | 0.36 (0.01) | 0.33 (0.04) | 0.78 (0.03) | 0.63 (0.03) | 0.68 (0.05) | 0.97 (0.01) | 1.10 (0.03) | 0.79 (0.04) | 0.63 (0.02) | 0.76 (0.03) |
| RFOP (BA) | 0.78 (0.01) | 0.62 (0.02) | 0.64 (0.01) | 0.50 (0.02) | 0.47 (0.01) | 0.38 (0.01) | 0.34 (0.03) | 0.77 (0.03) | 0.72 (0.04) | 0.70 (0.04) | 0.95 (0.01) | 1.11 (0.05) | 0.78 (0.03) | 0.63 (0.02) | 0.74 (0.02) |
| RFOP (MAE) | 0.78 (0.01) | 0.62 (0.02) | 0.65 (0.01) | 0.50 (0.02) | 0.47 (0.01) | 0.38 (0.01) | 0.34 (0.03) | 0.77 (0.03) | 0.64 (0.02) | 0.70 (0.04) | 0.94 (0.02) | 1.06 (0.03) | 0.78 (0.03) | 0.63 (0.02) | 0.76 (0.02) |
| RFOP (QWK) | 0.79 (0.02) | 0.63 (0.02) | 0.65 (0.01) | 0.50 (0.02) | 0.47 (0.01) | 0.38 (0.01) | 0.34 (0.04) | 0.76 (0.03) | 0.66 (0.04) | 0.72 (0.04) | 0.95 (0.01) | 1.08 (0.05) | 0.78 (0.03) | 0.64 (0.01) | 0.75 (0.02) |
| SVMOP (BA) | 0.77 (0.02) | 0.61 (0.02) | 0.63 (0.01) | 0.51 (0.01) | 0.46 (0.01) | 0.39 (0.01) | 0.34 (0.04) | 0.76 (0.04) | 0.65 (0.03) | 0.66 (0.04) | 0.93 (0.03) | 1.03 (0.02) | 0.79 (0.03) | 0.64 (0.02) | 0.76 (0.02) |
| SVMOP (MAE) | 0.77 (0.02) | 0.61 (0.02) | 0.63 (0.01) | 0.51 (0.01) | 0.46 (0.01) | 0.39 (0.01) | 0.34 (0.04) | 0.76 (0.04) | 0.65 (0.03) | 0.66 (0.04) | 0.93 (0.03) | 1.03 (0.02) | 0.79 (0.03) | 0.64 (0.02) | 0.76 (0.02) |
| SVMOP (QWK) | 0.77 (0.02) | 0.61 (0.02) | 0.63 (0.01) | 0.51 (0.02) | 0.46 (0.01) | 0.39 (0.01) | 0.35 (0.05) | 0.77 (0.04) | 0.65 (0.03) | 0.67 (0.06) | 0.94 (0.02) | 1.03 (0.02) | 0.78 (0.04) | 0.64 (0.02) | 0.76 (0.02) |
| **late stage (>182 DAI)** | | | |  |  |  |  |  |  |  |  |  |  |  |  |
| CLM (BA) | 0.77 (0.02) | 0.53 (0.04) | 0.54 (0.01) | 0.60 (0.04) | 0.42 (0.01) | 0.46 (0.02) | 0.27 (0.02) | 0.70 (0.03) | 0.62 (0.02) | 0.47 (0.04) | 0.88 (0.05) | 0.75 (0.06) | 0.80 (0.03) | 0.65 (0.01) | 0.85 (0.01) |
| CLM (MAE) | 0.76 (0.02) | 0.51 (0.02) | 0.55 (0.02) | 0.61 (0.03) | 0.43 (0.04) | 0.45 (0.03) | 0.28 (0.02) | 0.71 (0.02) | 0.62 (0.01) | 0.45 (0.03) | 0.83 (0.04) | 0.74 (0.06) | 0.79 (0.05) | 0.64 (0.02) | 0.85 (0.01) |
| CLM (QWK) | 0.80 (0.02) | 0.50 (0.02) | 0.55 (0.03) | 0.61 (0.04) | 0.38 (0.02) | 0.46 (0.02) | 0.24 (0.02) | 0.72 (0.02) | 0.63 (0.02) | 0.46 (0.03) | 0.90 (0.03) | 0.75 (0.07) | 0.82 (0.03) | 0.68 (0.02) | 0.86 (0.01) |
| Ordinal Forest (BA) | 0.85 (0.02) | 0.71 (0.01) | 0.73 (0.01) | 0.53 (0.02) | 0.47 (0.01) | 0.40 (0.02) | 0.22 (0.03) | 0.59 (0.01) | 0.49 (0.02) | 0.65 (0.03) | 0.97 (0.02) | 0.98 (0.04) | 0.79 (0.04) | 0.66 (0.01) | 0.84 (0.01) |
| Ordinal Forest (MAE) | 0.85 (0.01) | 0.71 (0.01) | 0.74 (0.01) | 0.54 (0.03) | 0.47 (0.01) | 0.40 (0.02) | 0.21 (0.03) | 0.59 (0.01) | 0.49 (0.02) | 0.62 (0.05) | 0.97 (0.02) | 1.00 (0.04) | 0.80 (0.04) | 0.66 (0.02) | 0.84 (0.01) |
| Ordinal Forest (QWK) | 0.85 (0.02) | 0.71 (0.02) | 0.74 (0.02) | 0.53 (0.02) | 0.47 (0.01) | 0.41 (0.02) | 0.22 (0.03) | 0.59 (0.01) | 0.48 (0.02) | 0.65 (0.05) | 0.97 (0.02) | 0.99 (0.03) | 0.79 (0.04) | 0.66 (0.01) | 0.85 (0.01) |
| RFOP (BA) | 0.84 (0.02) | 0.70 (0.02) | 0.72 (0.01) | 0.51 (0.04) | 0.46 (0.01) | 0.40 (0.02) | 0.23 (0.04) | 0.62 (0.04) | 0.53 (0.02) | 0.68 (0.08) | 0.99 (0.04) | 1.04 (0.08) | 0.78 (0.06) | 0.63 (0.03) | 0.84 (0.01) |
| RFOP (MAE) | 0.84 (0.02) | 0.71 (0.03) | 0.72 (0.03) | 0.51 (0.03) | 0.46 (0.02) | 0.40 (0.02) | 0.24 (0.03) | 0.60 (0.05) | 0.52 (0.03) | 0.68 (0.05) | 0.97 (0.04) | 1.03 (0.07) | 0.78 (0.05) | 0.66 (0.05) | 0.84 (0.01) |
| RFOP (QWK) | 0.84 (0.02) | 0.71 (0.02) | 0.72 (0.03) | 0.52 (0.02) | 0.46 (0.02) | 0.40 (0.02) | 0.25 (0.03) | 0.60 (0.05) | 0.52 (0.03) | 0.68 (0.03) | 0.99 (0.05) | 1.02 (0.06) | 0.78 (0.05) | 0.66 (0.04) | 0.84 (0.01) |
| SVMOP (BA) | 0.82 (0.02) | 0.62 (0.04) | 0.67 (0.01) | 0.49 (0.02) | 0.42 (0.03) | 0.36 (0.02) | 0.27 (0.03) | 0.74 (0.07) | 0.60 (0.03) | 0.73 (0.05) | 1.03 (0.07) | 1.07 (0.10) | 0.73 (0.04) | 0.58 (0.04) | 0.80 (0.02) |
| SVMOP (MAE) | 0.82 (0.02) | 0.65 (0.01) | 0.67 (0.01) | 0.48 (0.02) | 0.44 (0.01) | 0.36 (0.02) | 0.28 (0.03) | 0.69 (0.04) | 0.61 (0.02) | 0.74 (0.06) | 1.00 (0.07) | 1.07 (0.10) | 0.71 (0.05) | 0.61 (0.03) | 0.80 (0.01) |
| SVMOP (QWK) | 0.82 (0.02) | 0.65 (0.01) | 0.67 (0.01) | 0.48 (0.02) | 0.44 (0.01) | 0.36 (0.02) | 0.28 (0.03) | 0.69 (0.04) | 0.60 (0.02) | 0.74 (0.06) | 1.00 (0.07) | 1.06 (0.10) | 0.71 (0.05) | 0.61 (0.03) | 0.80 (0.01) |


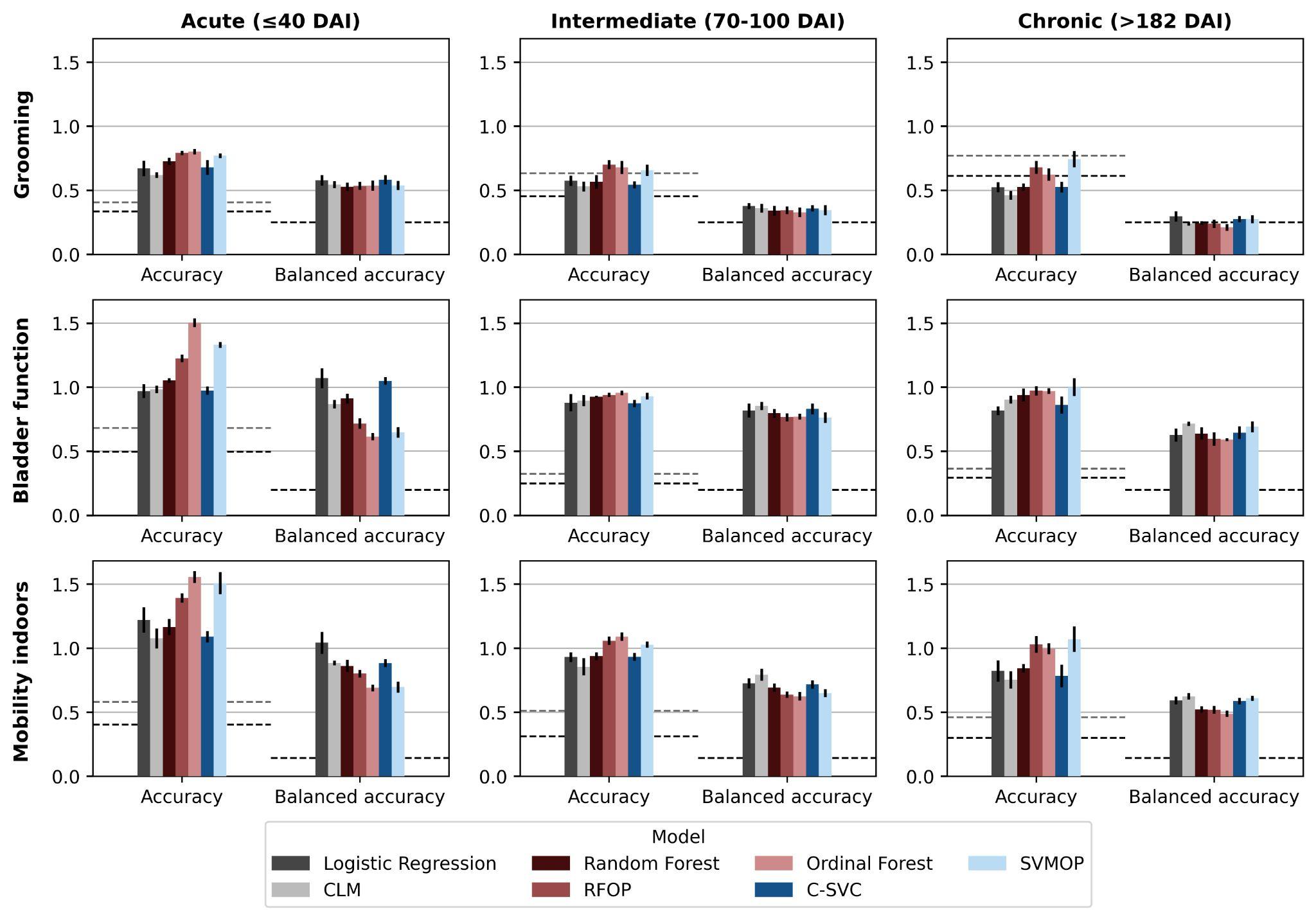


**Figure S2:** MAE and macro-MAE of all models. Mean and standard deviation over the five cross-validation folds are displayed. Columns represent the data subsets and rows show the outcomes. DAI, Days after injury; CLM, Cumulative link model; RFOP, Random Forest with ordered partitions; C-SVC, C-Support Vector Classification; SVMOP, Support Vector Classification with ordered partitions; CNN, Convolutional Neural Network.


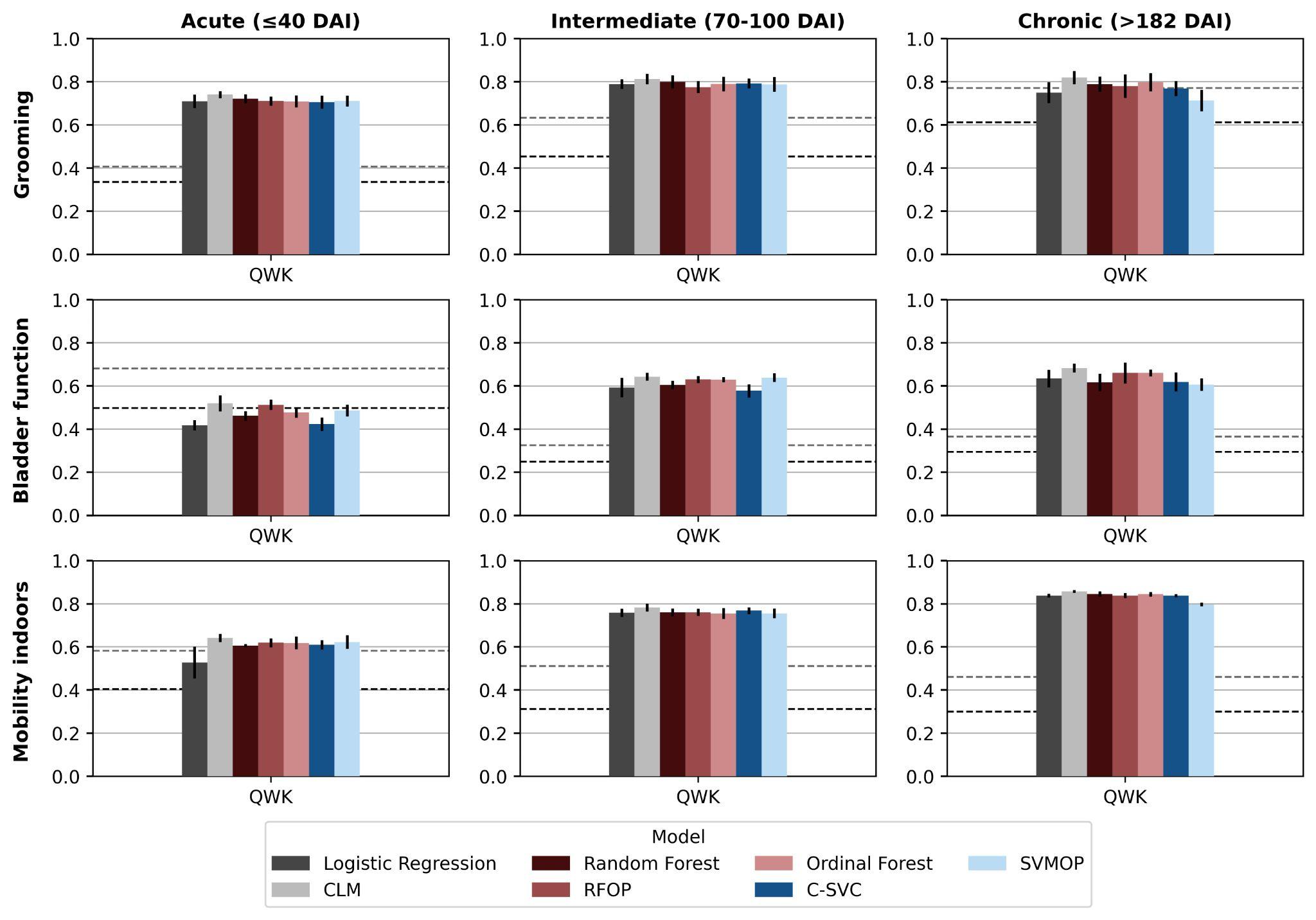


**Figure S3:** QWK of all models. Mean and standard deviation over the five cross-validation folds are displayed. Columns represent the data subsets and rows show the outcomes. DAI, Days after injury; CLM, Cumulative link model; RFOP, Random Forest with ordered partitions; C-SVC, C-Support Vector Classification; SVMOP, Support Vector Classification with ordered partitions; CNN, Convolutional Neural Network.

###

### Confusion matrices


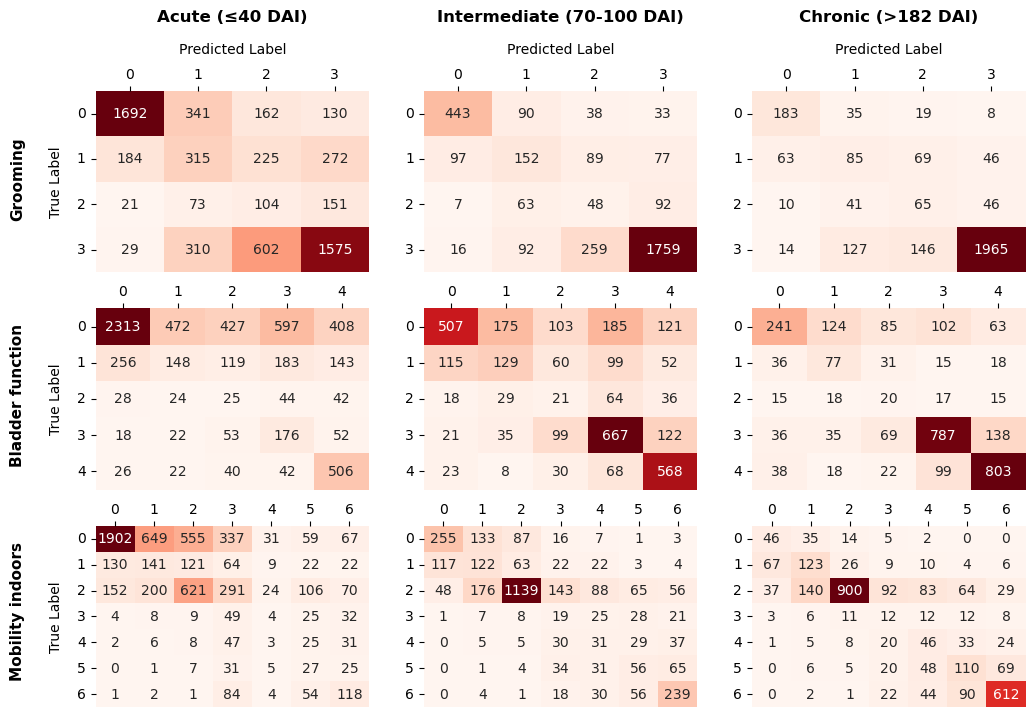
**Figure S4:** Confusion matrix of the Logistic Regression model.


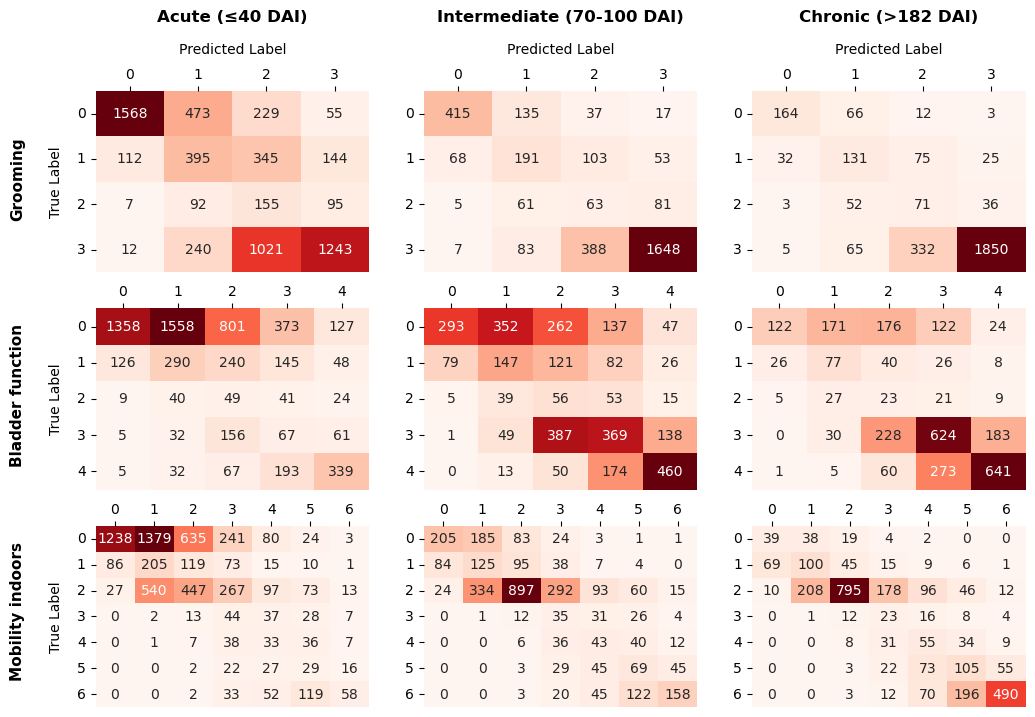


**Figure S5:** Confusion matrix of the Cumulative link model**.**


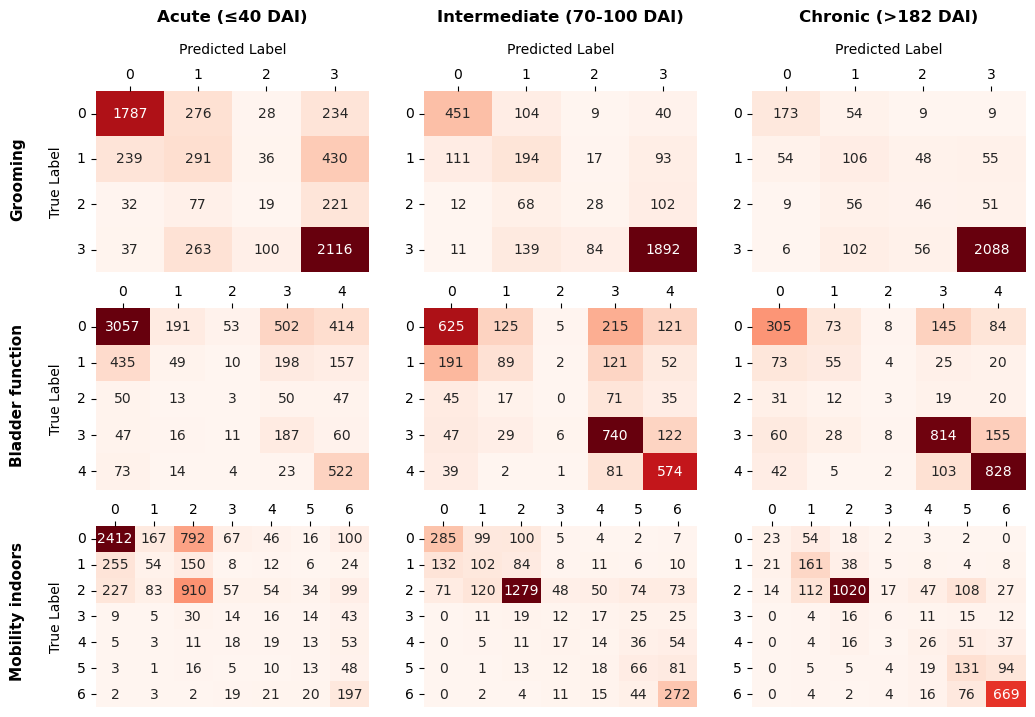


**Figure S6:** Confusion matrix of the Random Forest model.


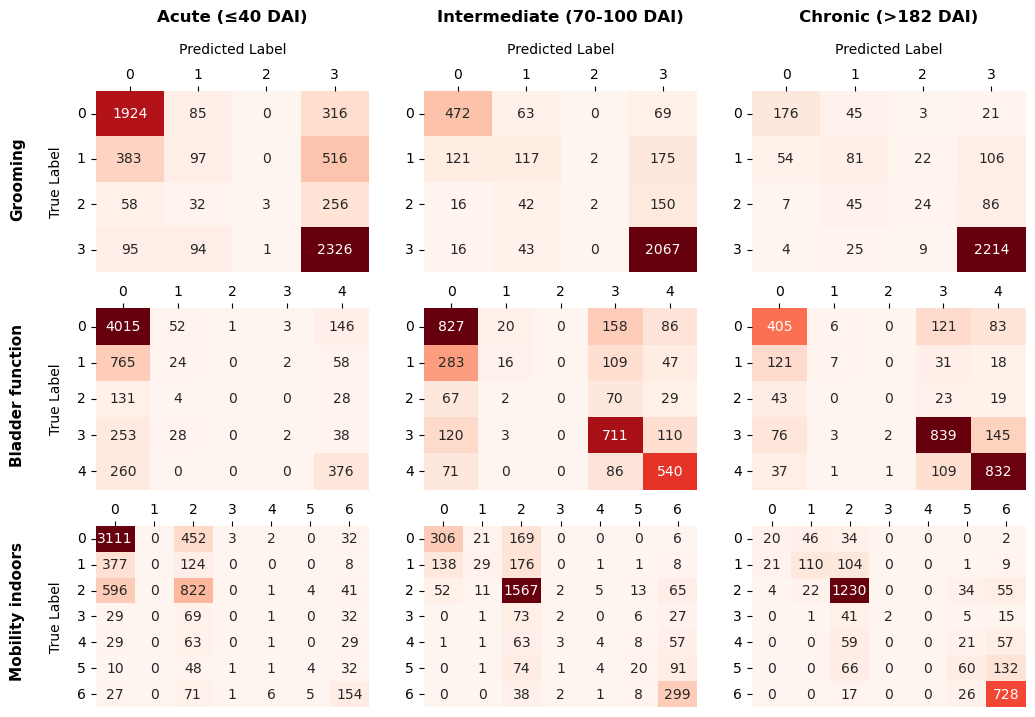


**Figure S7:** Confusion matrix of the Ordinal Forest model.


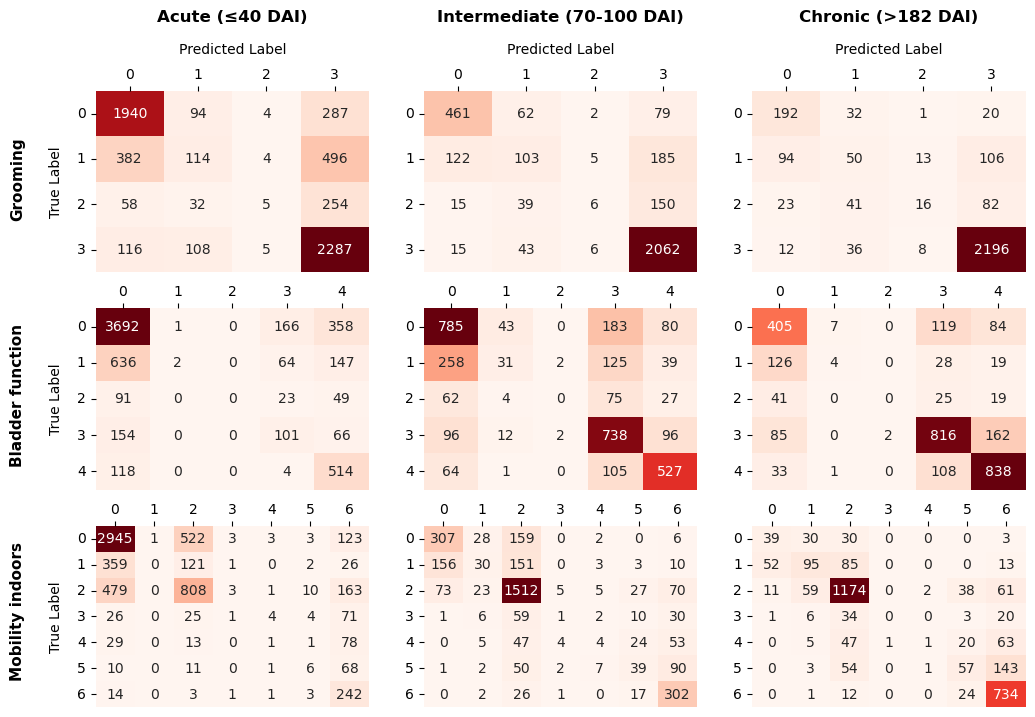


**Figure S8:** Confusion matrix of the ordered partitions model with a Random Forest as base estimator.


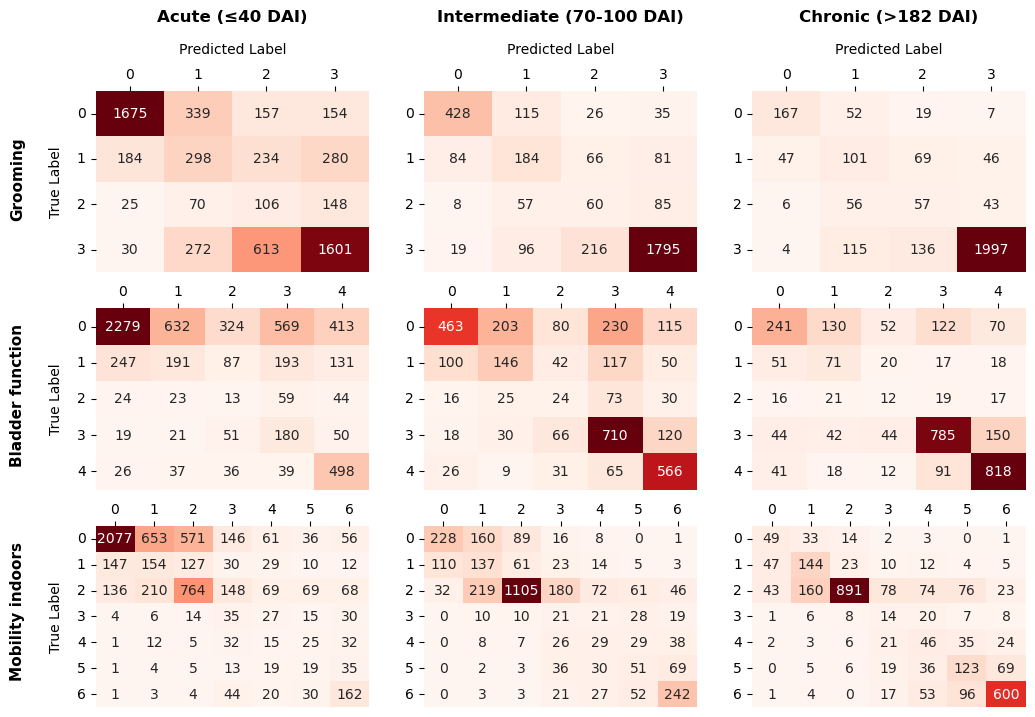


**Figure S9:** Confusion matrix of the C-Support Vector Classification model.


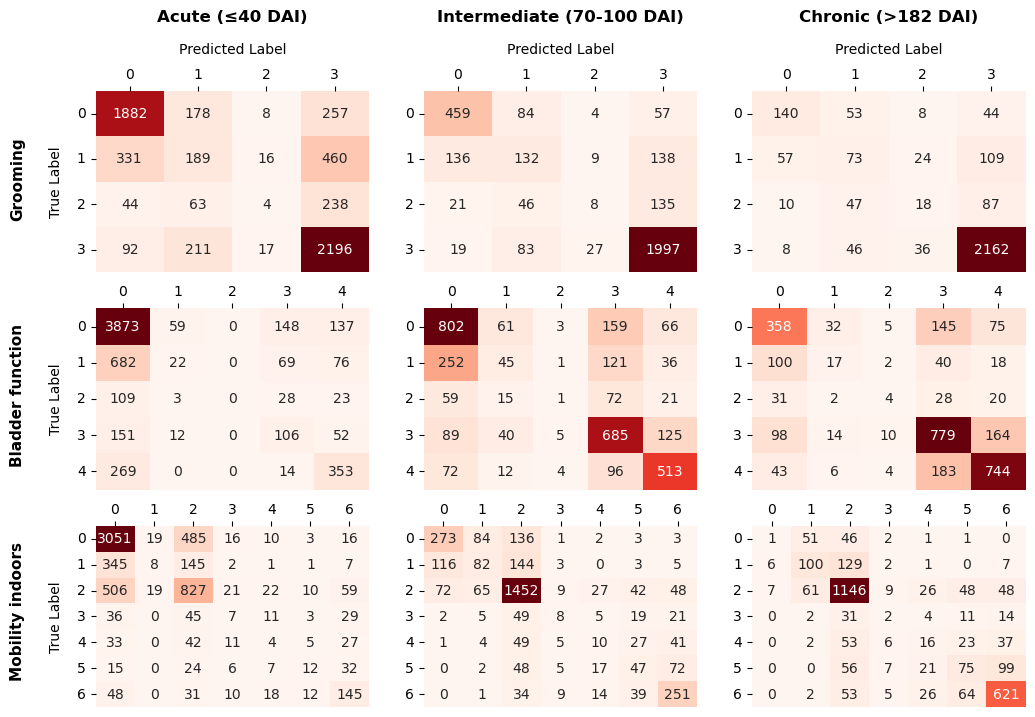


**Figure S10:** Confusion matrix of the ordered partitions model with a C-Support Vector Classification as base estimator.

###

### Feature importance


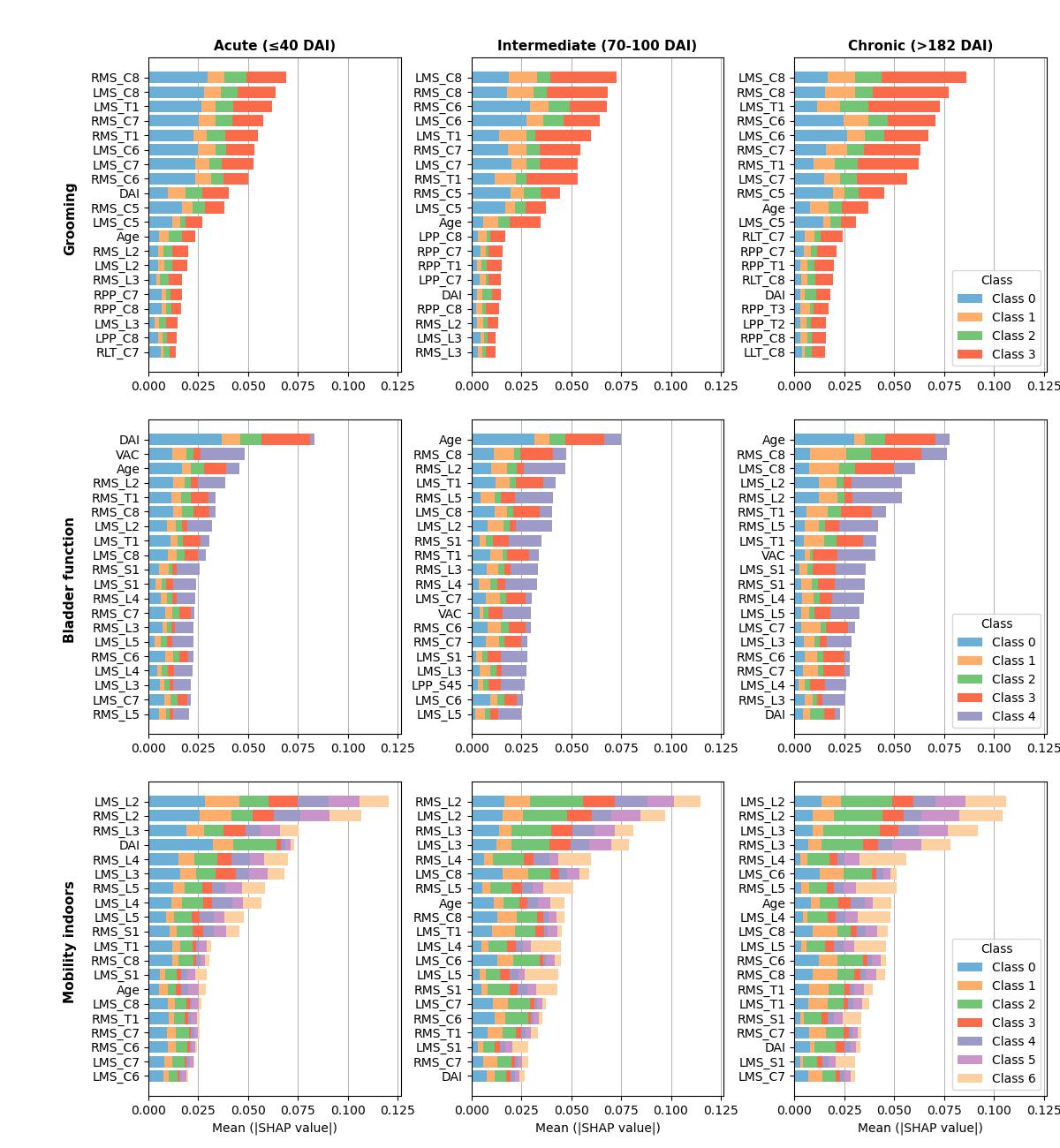


**Figure S11:** Mean absolute SHAP values stacked by classes. Columns represent the data subsets and rows show the outcomes. SHAP, Shapley additive explanations; DAI, Days after injury; RMS, right motor score, LMS, left motor score, RPP, right pin prick, LPP, left pin prick; RLT, right light touch, LLT left light touch, VAC, voluntary anal contractions.


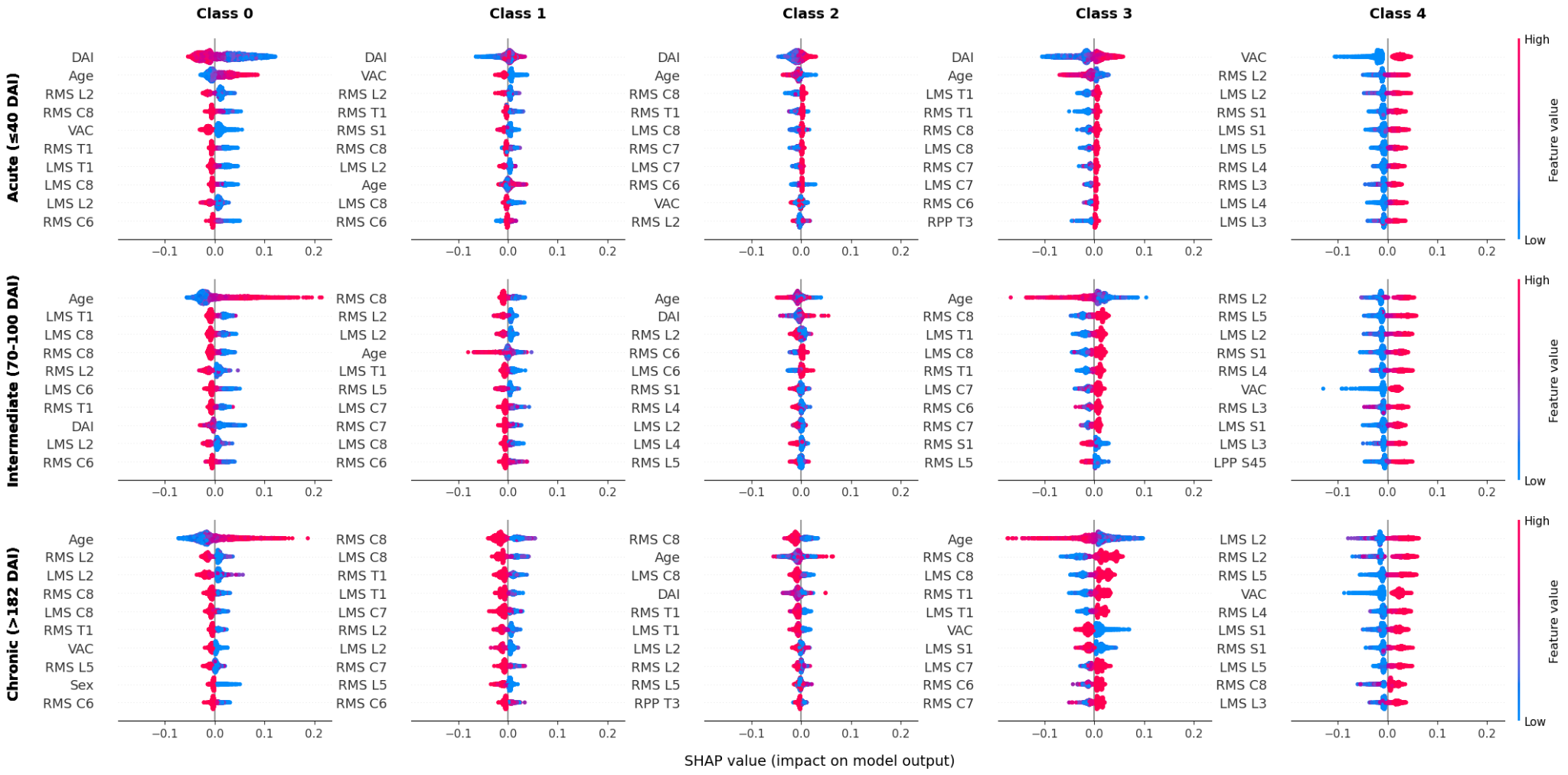


**Figure S12:** Raw SHAP values of the ten most important features for each class of SCIM item 6: Sphincter Management - Bladder. Features are sorted by the highest mean absolute SHAP value in descending order and data points are coloured by feature value. Rows show the data subsets and columns the classes. For sex, 1 refers to male and 0 to female. SHAP, Shapley additive explanations; SCIM, Spinal Cord Independence Measure; DAI, Days after injury; RMS, right motor score, LMS, left motor score.


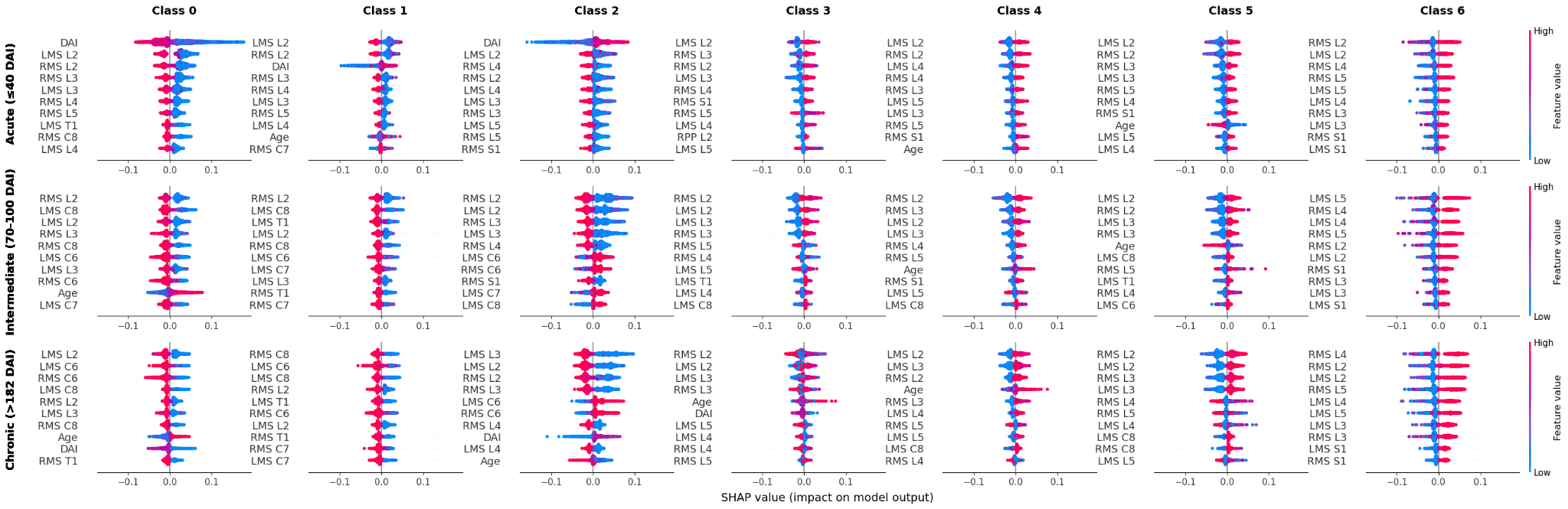


**Figure S13:** Raw SHAP values of the ten most important features for each class of SCIM item 12: Mobility indoors. Features are sorted by the highest mean absolute SHAP value in descending order and data points are coloured by feature value. Rows show the data subsets and columns the classes. SHAP, Shapley additive explanations; SCIM, Spinal Cord Independence Measure; DAI, Days after injury; RMS, right motor score, LMS, left motor score.


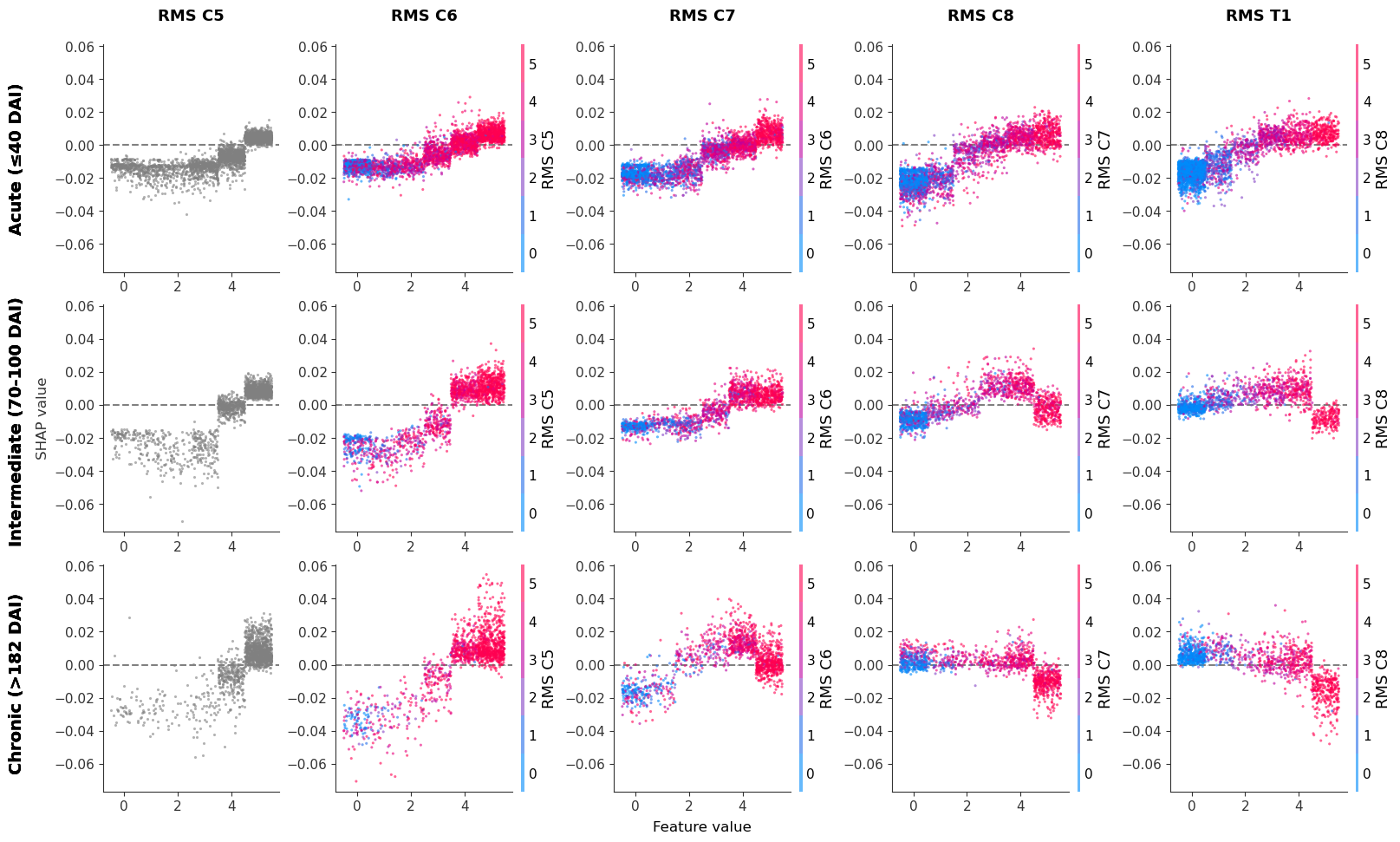


**Figure S14:** Raw SHAP values of the right motor scores C5 to T1 for class 0 of SCIM item 4: Grooming in tetraplegic patients. Data points of segments C6 to T1 are coloured by the segment that lies cranial to them. Rows show the data subsets and columns the motor scores of the different spinal levels. SHAP, Shapley additive explanations; SCIM, Spinal Cord Independence Measure; DAI, Days after injury; RMS, right motor score.


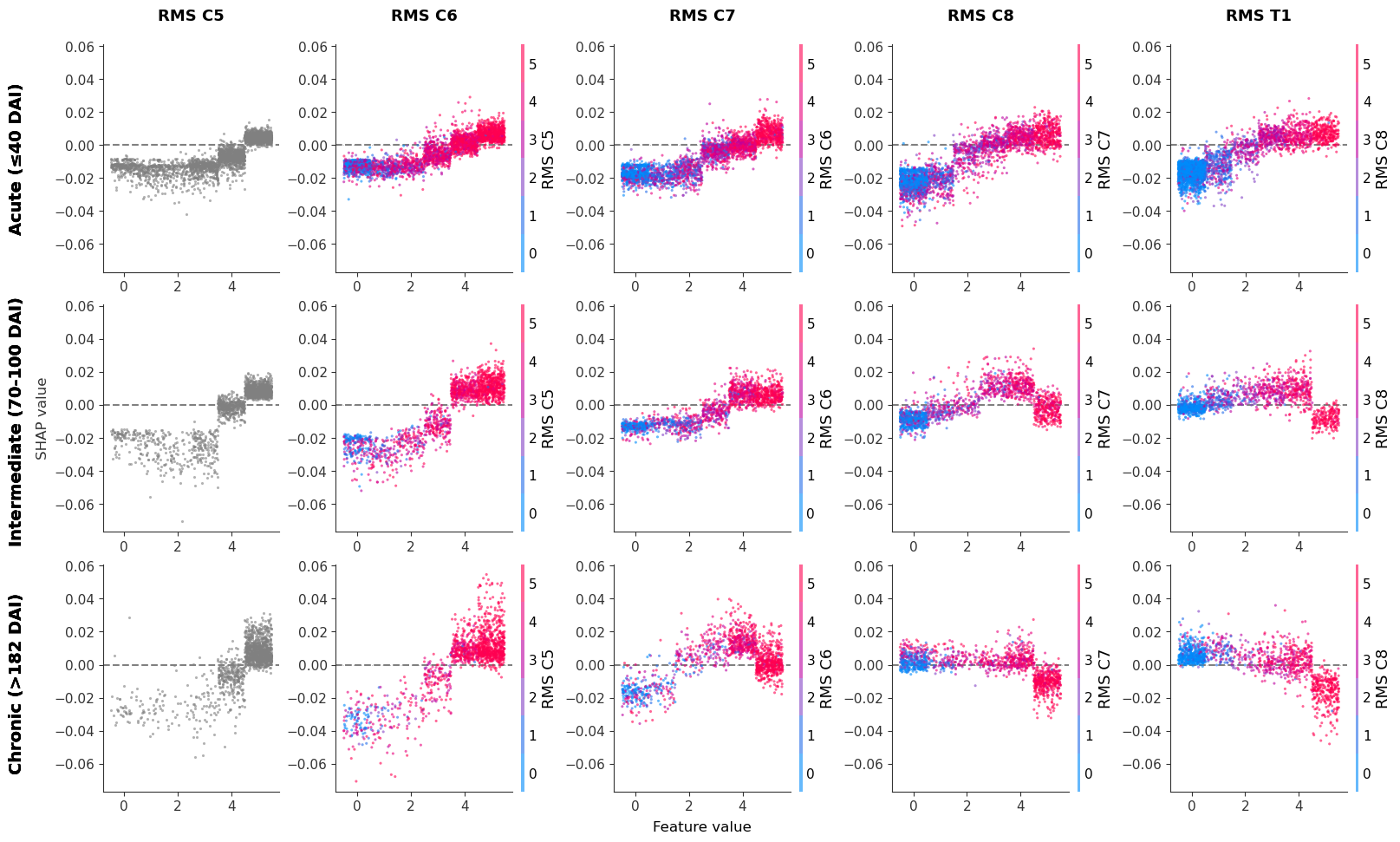


**Figure S15:** Raw SHAP values of the right motor scores C5 to T1 for class 2 of SCIM item 4: Grooming in tetraplegic patients. Data points of segments C6 to T1 are coloured by the segment that lies cranial to them. Rows show the data subsets and columns the motor scores of the different spinal levels. SHAP, Shapley additive explanations; SCIM, Spinal Cord Independence Measure; DAI, Days after injury; RMS, right motor score.


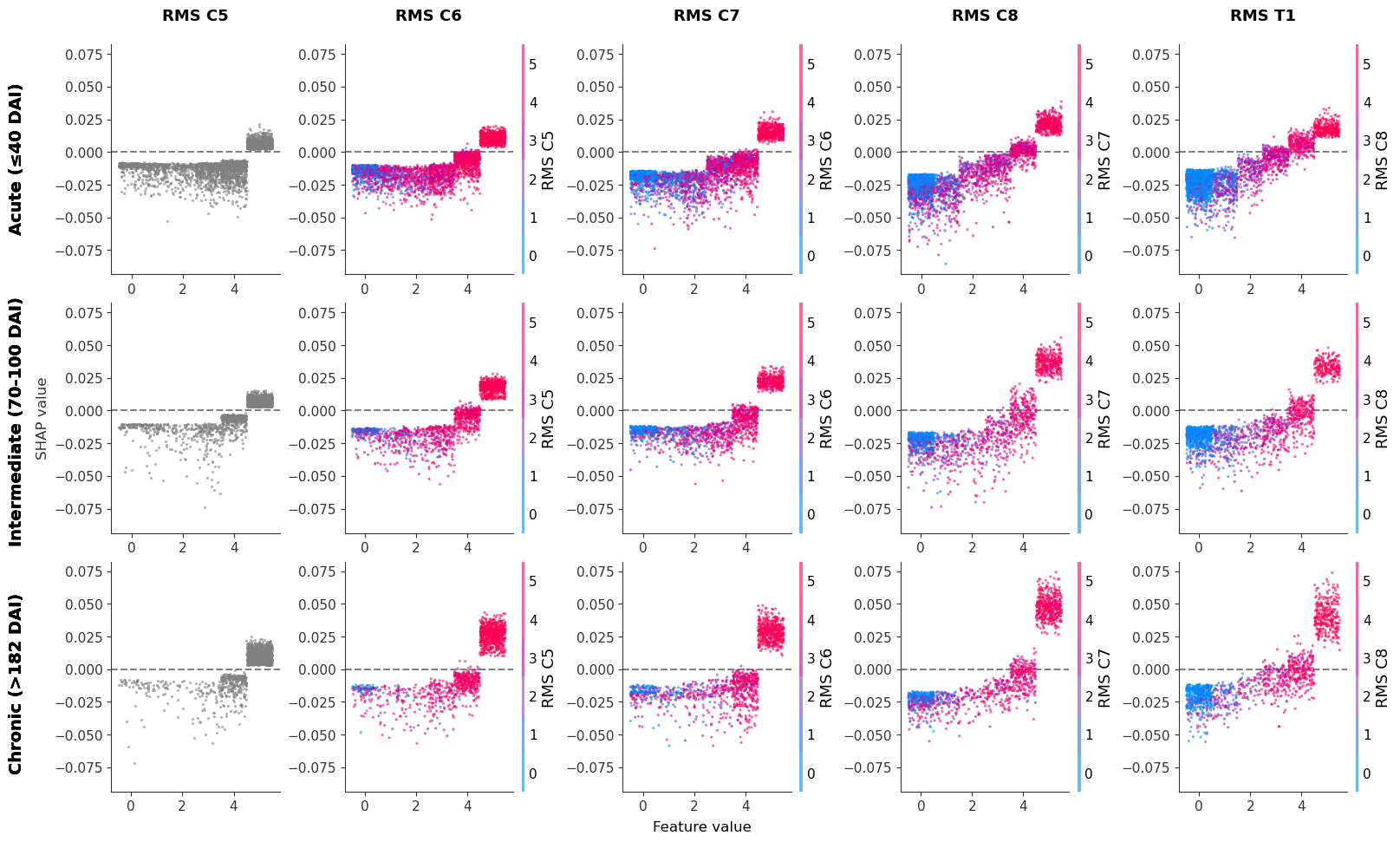


**Figure S16:**  Raw SHAP values of the right motor scores C5 to T1 for class 3 of SCIM item 4: Grooming in tetraplegic patients. Data points of segments C6 to T1 are coloured by the segment that lies cranial to them. Rows show the data subsets and columns the motor scores of the different spinal levels. SHAP, Shapley additive explanations; SCIM, Spinal Cord Independence Measure; DAI, Days after injury; RMS, right motor score.

##

## Supplementary results for reduced input version

The results of the reduced input version using right and left upper and lower extremity motor score, left and right light touch, and pin prick score instead of all individual ISNCSCI motor and sensory scores are presented in this subsection.

### Model performance

**Table S7:** **Mean and standard deviation of relative performance scores averaged over all scenarios (i.e. data subset–outcome combinations).** Relative performance was determined relative to the best model in five different metrics (accuracy, balanced accuracy, mean absolute error, macro-averaged mean absolute error and quadratic weighted kappa). All metrics were combined using a simple additive weighting approach with equal weights.

| Model | Mean (std) |
| --- | --- |
| Random Forest | 0.9447 (0.025) |
| RFOP (QWK) | 0.9195 (0.021) |
| RFOP | 0.9193 (0.021) |
| RFOP (BA) | 0.9158 (0.023) |
| C-SVC | 0.9122 (0.044) |
| Logistic Regression | 0.912 (0.0912) |
| Ordinal Forest (BA) | 0.909 (0.0309) |
| Ordinal Forest | 0.909 (0.030) |
| Ordinal Forest (QWK) | 0.9084 (0.028) |
| CLM | 0.8966 (0.058) |
| Regularized CLM (BA) | 0.8797 (0.061) |
| Regularized CLM (MAE) | 0.8635 (0.063) |
| SVMOP | 0.7743 (0.073) |
| SVMOP (QWK) | 0.7741 (0.074) |
| SVMOP (BA) | 0.7735 (0.075) |

**Table S8:** Results of all models in five different evaluation metrics. Mean and standard deviation over the 5 cross-validation folds are displayed. The best model(s) within each metric is marked in bold. DAI, Days after injury; QWK, quadratic weighted kappa; MAE, mean absolute error, Macro-MAE, macro-averaged mean absolute error; RFOP, Random Forest with ordered partitions; C-SVC, C-Support Vector Classification; CLM, Cumulative link model; SVMOP, Support Vector Classification with ordered partitions; CNN, Convolutional Neural Network.

|  | Grooming | Bladder function | Mobility indoors | Grooming | Bladder function | Mobility indoors | Grooming | Bladder function | Mobility indoors | Grooming | Bladder function | Mobility indoors | Grooming | Bladder function | Mobility indoors |
| --- | --- | --- | --- | --- | --- | --- | --- | --- | --- | --- | --- | --- | --- | --- | --- |
|  | Accuracy | | | Balanced Accuracy | | | MAE | | | Macro averaged MAE | | | QWK | | |
| **Acute stage (≤40 DAI)** | | | |  | | |  | | |  | | |  | | |
| Logistic Regression | 0.62 (0.01) | 0.54 (0.01) | 0.48 (0.08) | 0.50 (0.02) | 0.45 (0.01) | 0.35 (0.04) | 0.55 (0.03) | 1.04 (0.05) | 1.06 (0.17) | 0.66 (0.03) | 0.96 (0.03) | 1.21 (0.11) | 0.72 (0.02) | 0.43 (0.03) | 0.52 (0.08) |
| CLM | 0.61 (0.03) | 0.51 (0.02) | 0.47 (0.02) | 0.50 (0.01) | 0.37 (0.02) | 0.32 (0.04) | 0.54 (0.03) | 0.88 (0.03) | 0.90 (0.02) | 0.63 (0.02) | 0.99 (0.05) | 1.08 (0.06) | 0.74 (0.02) | 0.52 (0.04) | 0.64 (0.02) |
| Random Forest | 0.66 (0.01) | 0.63 (0.01) | 0.60 (0.02) | 0.49 (0.01) | 0.45 (0.01) | 0.37 (0.03) | 0.53 (0.02) | 0.84 (0.03) | 0.80 (0.03) | 0.71 (0.02) | 1.02 (0.04) | 1.13 (0.08) | 0.72 (0.02) | 0.49 (0.02) | 0.63 (0.01) |
| RFOP | 0.69 (0.01) | 0.69 (0.01) | 0.65 (0.01) | 0.47 (0.01) | 0.42 (0.02) | 0.35 (0.02) | 0.55 (0.04) | 0.72 (0.02) | 0.76 (0.03) | 0.79 (0.02) | 1.15 (0.06) | 1.36 (0.06) | 0.70 (0.03) | 0.52 (0.03) | 0.64 (0.02) |
| Ordinal Forest | 0.70 (0.02) | 0.71 (0.01) | 0.66 (0.01) | 0.47 (0.01) | 0.33 (0.01) | 0.29 (0.01) | 0.54 (0.04) | 0.62 (0.03) | 0.71 (0.02) | 0.80 (0.02) | 1.45 (0.02) | 1.57 (0.07) | 0.71 (0.03) | 0.49 (0.03) | 0.61 (0.03) |
| C-SVC | 0.60 (0.02) | 0.53 (0.02) | 0.52 (0.02) | 0.49 (0.02) | 0.45 (0.02) | 0.37 (0.03) | 0.57 (0.04) | 1.05 (0.04) | 0.94 (0.06) | 0.67 (0.05) | 0.97 (0.05) | 1.10 (0.09) | 0.71 (0.03) | 0.43 (0.01) | 0.59 (0.02) |
| SVMOP | 0.66 (0.01) | 0.69 (0.01) | 0.63 (0.02) | 0.45 (0.01) | 0.31 (0.01) | 0.28 (0.02) | 0.61 (0.03) | 0.71 (0.04) | 0.78 (0.04) | 0.83 (0.02) | 1.52 (0.03) | 1.81 (0.07) | 0.66 (0.03) | 0.36 (0.03) | 0.52 (0.02) |
| **Intermediate stage (70-100 DAI)** | | | |  |  |  |  |  |  |  |  |  |  |  |  |
| Logistic Regression | 0.72 (0.02) | 0.58 (0.01) | 0.56 (0.01) | 0.55 (0.03) | 0.49 (0.01) | 0.44 (0.02) | 0.36 (0.03) | 0.77 (0.03) | 0.74 (0.04) | 0.55 (0.04) | 0.86 (0.05) | 0.92 (0.07) | 0.81 (0.02) | 0.63 (0.02) | 0.76 (0.02) |
| CLM | 0.72 (0.02) | 0.45 (0.02) | 0.46 (0.02) | 0.56 (0.03) | 0.40 (0.03) | 0.40 (0.02) | 0.34 (0.03) | 0.85 (0.03) | 0.77 (0.03) | 0.53 (0.04) | 0.89 (0.06) | 0.85 (0.03) | 0.82 (0.03) | 0.66 (0.02) | 0.78 (0.01) |
| Random Forest | 0.78 (0.02) | 0.61 (0.01) | 0.60 (0.01) | 0.54 (0.04) | 0.49 (0.01) | 0.43 (0.02) | 0.33 (0.04) | 0.77 (0.03) | 0.70 (0.03) | 0.59 (0.06) | 0.90 (0.03) | 0.93 (0.05) | 0.81 (0.03) | 0.62 (0.02) | 0.76 (0.02) |
| RFOP | 0.79 (0.02) | 0.62 (0.02) | 0.64 (0.01) | 0.52 (0.03) | 0.47 (0.01) | 0.38 (0.02) | 0.33 (0.04) | 0.75 (0.04) | 0.66 (0.05) | 0.67 (0.05) | 0.93 (0.02) | 1.08 (0.06) | 0.79 (0.03) | 0.64 (0.02) | 0.75 (0.02) |
| Ordinal Forest | 0.79 (0.02) | 0.62 (0.01) | 0.65 (0.01) | 0.51 (0.03) | 0.47 (0.01) | 0.37 (0.01) | 0.33 (0.04) | 0.77 (0.04) | 0.64 (0.05) | 0.67 (0.05) | 0.94 (0.03) | 1.10 (0.03) | 0.79 (0.03) | 0.63 (0.03) | 0.75 (0.03) |
| C-SVC | 0.74 (0.02) | 0.57 (0.01) | 0.54 (0.02) | 0.55 (0.02) | 0.49 (0.02) | 0.42 (0.03) | 0.35 (0.03) | 0.78 (0.04) | 0.75 (0.03) | 0.56 (0.03) | 0.85 (0.05) | 0.92 (0.03) | 0.81 (0.02) | 0.63 (0.03) | 0.75 (0.02) |
| SVMOP | 0.75 (0.02) | 0.56 (0.01) | 0.59 (0.02) | 0.48 (0.03) | 0.42 (0.01) | 0.34 (0.02) | 0.43 (0.05) | 0.91 (0.02) | 0.75 (0.05) | 0.78 (0.07) | 1.07 (0.01) | 1.15 (0.07) | 0.69 (0.05) | 0.55 (0.01) | 0.68 (0.05) |
| **late stage (>182 DAI)** | | | |  |  |  |  |  |  |  |  |  |  |  |  |
| Logistic Regression | 0.79 (0.01) | 0.67 (0.02) | 0.62 (0.02) | 0.61 (0.03) | 0.52 (0.01) | 0.47 (0.03) | 0.28 (0.02) | 0.62 (0.04) | 0.61 (0.03) | 0.48 (0.05) | 0.82 (0.04) | 0.83 (0.05) | 0.77 (0.04) | 0.64 (0.04) | 0.84 (0.01) |
| CLM | 0.80 (0.01) | 0.49 (0.02) | 0.53 (0.02) | 0.61 (0.03) | 0.38 (0.02) | 0.44 (0.04) | 0.24 (0.01) | 0.73 (0.02) | 0.64 (0.03) | 0.46 (0.03) | 0.91 (0.04) | 0.77 (0.07) | 0.83 (0.03) | 0.69 (0.03) | 0.85 (0.01) |
| Random Forest | 0.83 (0.02) | 0.69 (0.01) | 0.68 (0.02) | 0.61 (0.03) | 0.50 (0.01) | 0.46 (0.02) | 0.23 (0.02) | 0.61 (0.03) | 0.55 (0.03) | 0.49 (0.04) | 0.88 (0.03) | 0.86 (0.04) | 0.80 (0.04) | 0.65 (0.03) | 0.84 (0.01) |
| RFOP | 0.84 (0.02) | 0.70 (0.02) | 0.72 (0.01) | 0.52 (0.02) | 0.46 (0.02) | 0.40 (0.01) | 0.24 (0.04) | 0.62 (0.04) | 0.51 (0.03) | 0.66 (0.03) | 0.97 (0.06) | 1.01 (0.08) | 0.78 (0.06) | 0.64 (0.03) | 0.84 (0.02) |
| Ordinal Forest | 0.85 (0.02) | 0.70 (0.01) | 0.72 (0.01) | 0.54 (0.03) | 0.47 (0.01) | 0.39 (0.01) | 0.23 (0.03) | 0.61 (0.01) | 0.52 (0.01) | 0.63 (0.04) | 0.97 (0.04) | 1.02 (0.07) | 0.78 (0.04) | 0.65 (0.01) | 0.84 (0.01) |
| C-SVC | 0.80 (0.02) | 0.65 (0.03) | 0.62 (0.02) | 0.61 (0.02) | 0.50 (0.02) | 0.48 (0.04) | 0.27 (0.03) | 0.64 (0.07) | 0.62 (0.02) | 0.49 (0.02) | 0.85 (0.03) | 0.82 (0.06) | 0.77 (0.05) | 0.63 (0.06) | 0.84 (0.01) |
| SVMOP | 0.79 (0.02) | 0.57 (0.01) | 0.63 (0.01) | 0.38 (0.02) | 0.37 (0.02) | 0.30 (0.02) | 0.38 (0.04) | 0.85 (0.03) | 0.76 (0.05) | 1.07 (0.04) | 1.15 (0.07) | 1.23 (0.09) | 0.47 (0.04) | 0.46 (0.02) | 0.71 (0.03) |

### Feature importance


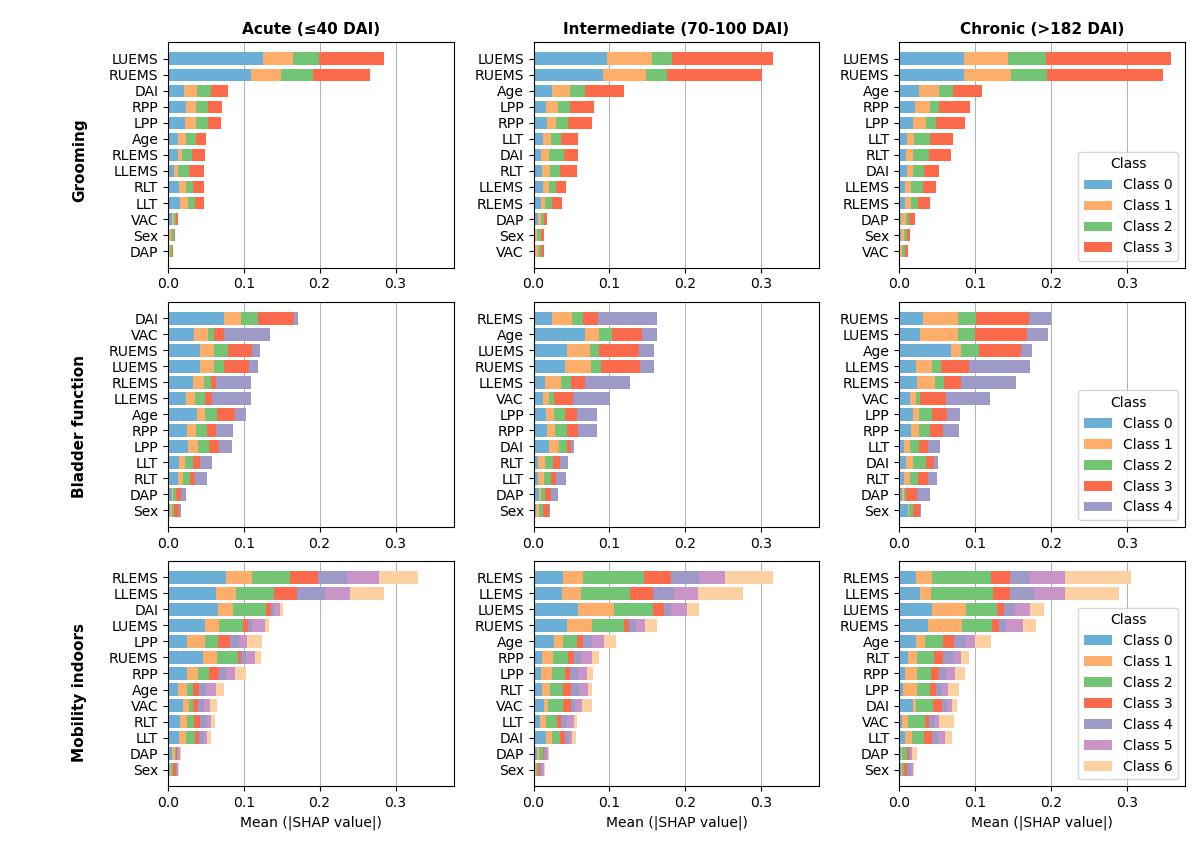


**Figure S17:** Mean absolute SHAP values stacked by classes. Columns represent the data subsets and rows show the outcomes. SHAP, Shapley additive explanations; DAI, Days after injury; RUEMS, right upper extremity motor score; LUEMS, left upper extremity motor score; RLEMS, right lower extremity motor score, LLEMS, left lower extremity motor score; RPP, right pin prick; LPP, left pin prick; RLT, right light touch; LLT, left light touch; VAC, voluntary anal contractions; DAP, deep anal pressure.


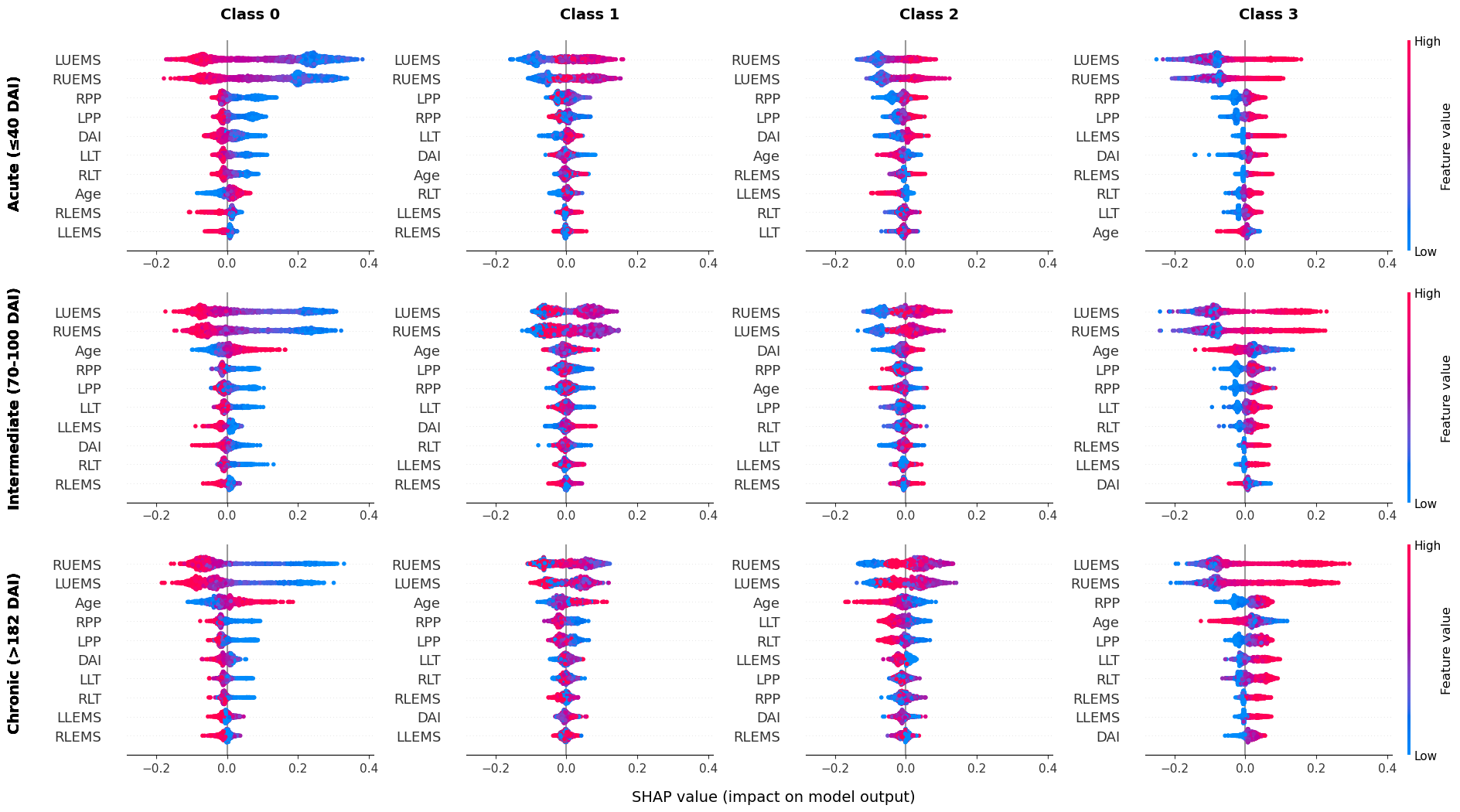


**Figure S18:** Raw SHAP values of the ten most important features for each class of SCIM item 4: Grooming in tetraplegic patients. Features are sorted by the highest mean absolute SHAP value in descending order and data points are coloured by feature value. Rows show the data subsets and columns the classes. SHAP, Shapley additive explanations; SCIM, Spinal Cord Independence Measure; DAI, Days after injury; RUEMS, right upper extremity motor score; LUEMS, left upper extremity motor score; RLEMS, right lower extremity motor score, LLEMS, left lower extremity motor score; RPP, right pin prick; LPP, left pin prick; RLT, right light touch; LLT, left light touch; VAC, voluntary anal contractions; DAP, deep anal pressure.


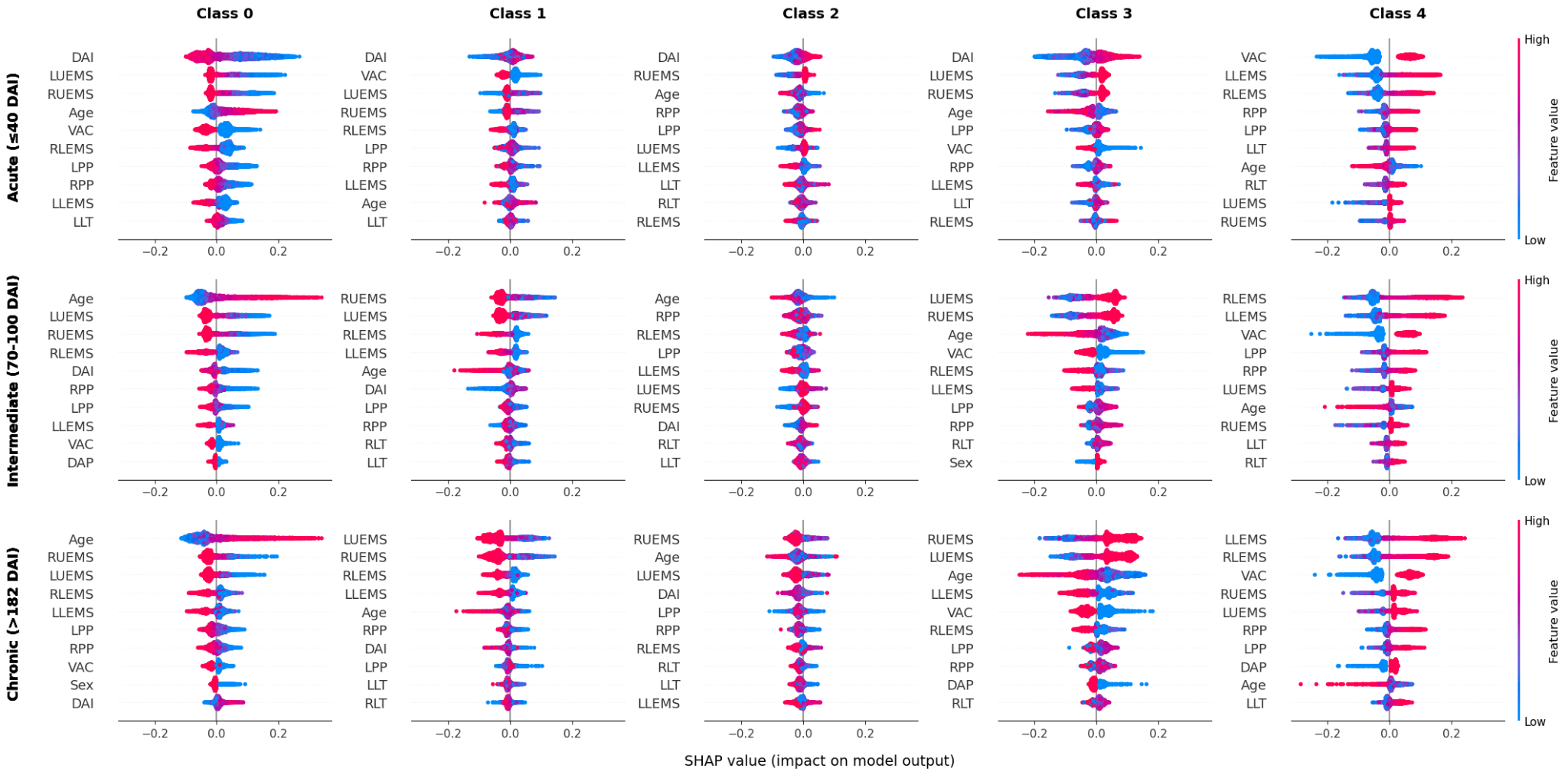


**Figure S19:** Raw SHAP values of the ten most important features for each class of SCIM item 6: Sphincter Management - Bladder. Features are sorted by the highest mean absolute SHAP value in descending order and data points are coloured by feature value. Rows show the data subsets and columns the classes. SHAP, Shapley additive explanations; SCIM, Spinal Cord Independence Measure; DAI, Days after injury; RUEMS, right upper extremity motor score; LUEMS, left upper extremity motor score; RLEMS, right lower extremity motor score, LLEMS, left lower extremity motor score; RPP, right pin prick; LPP, left pin prick; RLT, right light touch; LLT, left light touch; VAC, voluntary anal contractions; DAP, deep anal pressure.


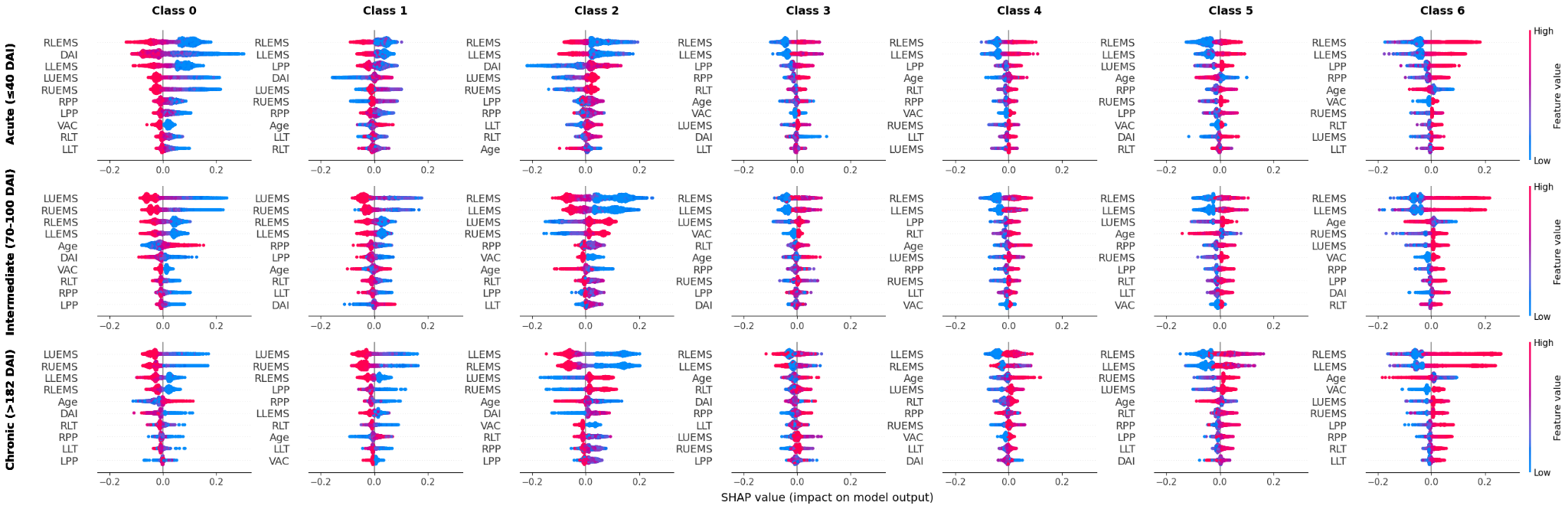


**Figure S20:** Raw SHAP values of the ten most important features for each class of SCIM item 12: Mobility indoors. Features are sorted by the highest mean absolute SHAP value in descending order and data points are coloured by feature value. Rows show the data subsets and columns the classes. SHAP, Shapley additive explanations; SCIM, Spinal Cord Independence Measure; DAI, Days after injury; RUEMS, right upper extremity motor score; LUEMS, left upper extremity motor score; RLEMS, right lower extremity motor score, LLEMS, left lower extremity motor score; RPP, right pin prick; LPP, left pin prick; RLT, right light touch; LLT, left light touch; VAC, voluntary anal contractions; DAP, deep anal pressure.
